# GMP‐Compliant Process for the Manufacturing of an Extracellular Vesicles‐Enriched Secretome Product Derived From Cardiovascular Progenitor Cells Suitable for a Phase I Clinical Trial

**DOI:** 10.1002/jev2.70145

**Published:** 2025-08-20

**Authors:** Camille Humbert, Chloé Cordier, Iouri Drut, Michele Hamrick, Jacquelyn Wong, Valérie Bellamy, Justine Flaire, Kiranmayee Bakshy, Florent Dingli, Damarys Loew, Jérôme Larghero, Jean‐Roch Fabreguettes, Philippe Menasché, Nisa K. Renault, Guillaume Churlaud

**Affiliations:** ^1^ Centre MEARY de Thérapie Cellulaire et Génique de l'Assistance Publique – Hôpitaux de Paris (AP‐HP) Direction de la Recherche Clinique et de l'Innovation (DRCI) Paris France; ^2^ FUJIFILM Cellular Dynamics, Inc. (FCDI) Madison Wisconsin USA; ^3^ Paris Centre de Recherche Cardiovasculaire (PARCC) Institut National de la Santé et de la Recherche Médicale (INSERM) Université Paris Cité Paris France; ^4^ Institut Curie Université Paris Sciences & Lettres (PSL) Centre de Recherche CurieCoreTech Spectrométrie de Masse Protéomique Paris France; ^5^ Université Paris Cité, INSERM Centre d'Investigation Clinique de Biothérapie (CIC‐BT) CBT501 Paris France; ^6^ Département Essais Cliniques, DRCI, AP‐HP Agence Générale des Equipements et Produits de Santé (AGEPS) Paris France; ^7^ Département de chirurgie cardiovasculaire Hôpital Européen Georges Pompidou (HEGP), AP‐HP Paris France

**Keywords:** extracellular vesicles (EV), GMP manufacturing, large‐scale process development, quality control, secretome

## Abstract

Extracellular vesicle (EV)‐enriched secretomes are emerging as a new and innovative therapeutic option in the field of regenerative medicine. The clinical use of EV‐enriched secretome‐based products requires manufacturing processes and quality control (QC) testing that comply with current good manufacturing practice (GMP). The goal of this work was to develop a robust and reproducible large‐scale GMP‐compliant process for the production of an EV‐enriched secretome derived from cardiovascular progenitor cells (CPC), including the vesiculation of CPC, purification and concentration of the product; and sterilising filtration. QC strategies for in‐process and release testing of an investigational medicinal product (IMP) were developed to guarantee quantity, safety, purity and identity. The IMP showed biological activity and was non‐immunogenic in vitro, and showed no signs of toxicity or tumour development in vivo. The IMP was approved for use in a single‐centre Phase I clinical trial by the French National Agency for Medicines and Health (*ANSM*) for the treatment of heart failure. The IMP is stored between –65°C and –85°C and can be easily diluted by the hospital pharmacy for infusion to the patient. This work represents a major advance for the use of CPC derived EV‐enriched secretomes as a biological drug for cardiac clinical applications.

**Trial Registration**: ClinicalTrials.gov identifier: NCT05774509

## 1 Introduction

Almost all cells secrete a wide variety of bioactive molecules such as proteins and bi‐lipid membrane‐bound, non‐replicative particles known as extracellular vesicles (EV) (Vlassov et al. 2012). The set of substances secreted by a cell, and not exclusively restricted to EV, can be defined as its secretome.

EV include several major sub‐types which are defined by their biological origin: exosomes (formed in the late endosome and exocytosed; ∼30–200 nm in size), microparticles/microvesicles/ectosomes (bud from the plasma membrane; ∼100–1000 nm) and apoptotic bodies (resulting from programmed cell death; >1000 nm). All EV have a complex composition, which is highly dependent on cell source, containing specific proteins and nucleic acids (Gurung et al. 2021). However, some markers have been identified as commonly present on most EV species: such is the case for the members of the tetraspanin family: CD9, CD63 and CD81 (Kowal et al. 2016; Ohno et al. 2016; Théry et al. 2018).

EV play a major role in intercellular communication (Sahoo and Losordo 2014; Yuan et al. 2018; Zeh et al. 2019; Mathieu et al. 2019) and, as such, are involved in various biological processes, including tissue regeneration and immune responses (Yáñez‐Mó et al. 2015). This involvement seems to stem from the ability of the EV and their cargo to activate tissue repair‐associated signalling pathways such as cell proliferation, angiogenesis, apoptosis and modulation of inflammation (Roefs et al. 2020). Therefore, EV have been studied for the treatment of several diseases and are already assessed in clinical trials for cancer, respiratory diseases and diabetes, while other potential indications are increasingly considered, in particular in the cardiovascular area (Lener et al. 2015; Barile and Vassalli 2017; Sluijter et al. 2018; Sanz‐Ros et al. 2023; Laura Francés et al. 2023).

Namely, in several animal models of cardiovascular disease, EV secreted by cardiovascular progenitor cells (CPC) have been shown to play a cardio‐protective role (Gray et al. 2015; Maring et al. 2019; Marbán and Liao 2022) and to exert therapeutic effects similar to cell therapies (Kervadec et al. 2016; El Harane et al. 2018; Barile et al. 2018). Proteins present in the secretome, in addition to the EV, also contribute to the cardioprotective effects (Torán et al. 2019; Yadid et al. 2020). While these studies are encouraging for the cardiac field, it is not clear whether the results from research‐grade secretome products, as described in these publications, are predictive of the therapeutic potential of their clinical‐grade counterparts.

Indeed, the manufacture of any Investigational Medicinal Product (IMP) is subject to good manufacturing practice (GMP) regulation, ensuring that production and testing meet established quality standards. To meet these standards, several switches will be required, such as changes from research use only (RUO) materials to xeno‐free or chemically defined GMP‐grade reagents having potentially different purity profiles or specific activities. Furthermore, the manufacturing process may require an upgrade from open to closed methods, and other procedural steps may likewise be changed for better manufacturability or process control at large scale (Gouveia et al. 2015). These changes can have small or dramatic effects on the therapeutic efficacy of the final product. This may be particularly so for EV‐based compositions, where the nature and growth conditions (including chemical or sheer stress) of the mother cells will affect the molecular composition of the EV; the EV isolation method will also greatly affect not only the profile of the isolated EV, but also the co‐isolated non‐EV components contained in the final composition (Ng et al. 2022; Alexandre et al. 2024).

A first challenge, is therefore, to lock down an EV‐enriched secretome manufacturing process which results in a final product that is GMP‐compliant, technically doable, therapeutically effective and safe for use in a Phase 1 clinical trial. Since the field of EV‐based therapeutics is in its infancy, a second major challenge is to develop a quality control (QC) release strategy which is meaningful, scientifically valid, acceptable to the regulatory agencies, and ensures that the manufacturing process is controlled and reproducible. In particular, the thorough characterisation of the molecular and functional parameters of the mother cells before and after vesiculation, and of the final cell‐free product, seems critical for enabling clinical applications.

We acknowledge that several teams have already proposed different methods for the development of a GMP‐compliant manufacturing method of EV‐based therapeutics (Lamparski et al. 2002; Andriolo et al. 2018; Bari et al. 2018; Busatto et al. 2018; Rohde et al. 2019; Laggner et al. 2020; Mocchi et al. 2021). However, our novel and proprietary, serum‐free, GMP‐ready method to generate, purify, isolate and enrich a secretome from CPC features several innovative aspects:

1. The use of a human induced pluripotent stem cell (hiPSC)‐derived cells as the source of the secretome. The initial starting point of every batch of product is, therefore, a single, well‐defined, and highly expandable source of cells. By starting from the same material, our process is therefore better poised to generate highly reproducible batches of clinical product. In previous work, EV were derived from primary cells (mesenchymal stem cell [MSC] or peripheral blood mononuclear cells [PBMC]) which rely on multiple donors over time, and are therefore subject to large inter‐individual variability in their starting materials and, consequently, final product lot to lot variability.
2. The use of scaled‐up tangential flow filtration (TFF) allowed for the processing of a large enough volume of conditioned media to cover the needs of QC release testing; Good Laboratory Practice (GLP) animal toxicity and tumorigenicity assessments; treatment of the patients for the whole clinical trial; and stability testing for more that 3 years. Of note, TFF was integrated into a fully closed method, uninterrupted until the IMP (final product) was obtained, with all methods, materials and reagents upgraded to GMP compatibility at the Phase I clinical manufacturing scale.
3. A QC strategy for in‐process and release testing that ensures the potency and safety of the final IMP and follows the recommendations of the Minimal Information for Studies of EV 2018 (MISEV2018) guidelines (Théry et al. 2018) and the regulations defined in the European Pharmacopoeia (EP).

In addition, the work described herein differs from many of the previously published methods in that it has been fully executed in GMP, the final product (considered a biological product) has been approved for use in a phase 1 clinical trial by the competent regulatory authority, and the IMP is being actively tested in humans in an ongoing clinical trial.

## 2 Materials and Methods

### 2.1 Manufacturing Process for an EV‐Enriched Secretome From CPC

#### 2.1.1 hiPSC‐Derived CPC

hiPSC‐derived CPC (‘FCDI CTC1’) were produced at the innovation Facility for Advanced Cell Therapy (iFACT, FUJIFILM Cellular Dynamics, Inc. (FCDI); Madison, Wi, USA). CPC generation was performed in a GMP suite using a novel differentiation process utilising GMP‐compatible methods, materials and reagents, at Phase I clinical manufacturing scale, according to a proprietary technology developed and owned by FCDI. Detailed methods were submitted to, reviewed by and approved by the French National Agency for Medicines and Health (*ANSM* = *Agence Nationale de Sécurité du Médicament et des produits de santé*). CPC cells were cryopreserved and shipped in liquid nitrogen vapour phase to France for further processing.

#### 2.1.2 EV‐Enriched Secretome From CPC

**FIGURE 1:**
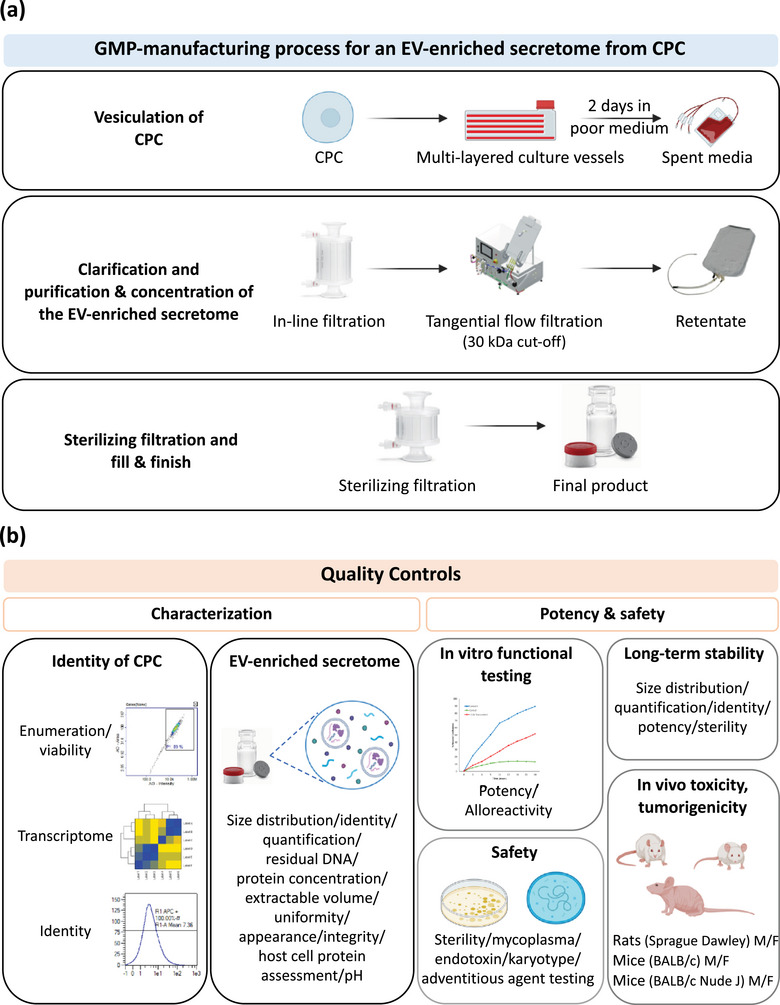
GMP‐manufacturing process for an EV‐enriched secretome from CPC. (a) Schematic representation of the three main steps of the GMP‐manufacturing of the EV‐enriched secretome final product. (b) Overview of the QC strategy for the characterisation of CPC during the manufacturing process (in‐process monitoring) and of the final product. M/F, Male/Female. Image created with BioRender.com.

All processes were conducted in accordance with GMP regulations at the *Centre MEARY de Thérapie Cellulaire et Génique de l'Assistance Publique—Hôpitaux de Paris* (AP‐HP) (EudraGMP authorisation for manufacturing and QC TIE/21/O/001) using GMP‐compatible methods, materials and reagents, at Phase I clinical manufacturing scale according to proprietary technology as described below (Figure 1a).

The final product was produced and formulated under aseptic conditions in a Class A space, in Class B cleanrooms. Class A is composed of Fan‐Filter Units (FFU). The TFF is placed under FFU to maintain aseptic conditions (Table 1).

**TABLE 1:** RUO versus GMP‐compatible grade. Setup of GMP‐compatible methods, materials and reagents, at Phase I clinical manufacturing scale.

		RUO	GMP‐compatible grade
Documentation		Technical operative methods	Quality assurance system, batch records Risk‐based approach to manufacturing (ICHQ9)
Raw materials		Data sheet and CoA (if available)	Robust controls, audit of suppliers, CoA, CoO, EC certificates
Equipment		General laboratory	Qualification and control, calibration, maintenance, validation
Human resources		General laboratory qualification	GMP and aseptic qualification
Facility		Class A laminar hood in non‐classified environment 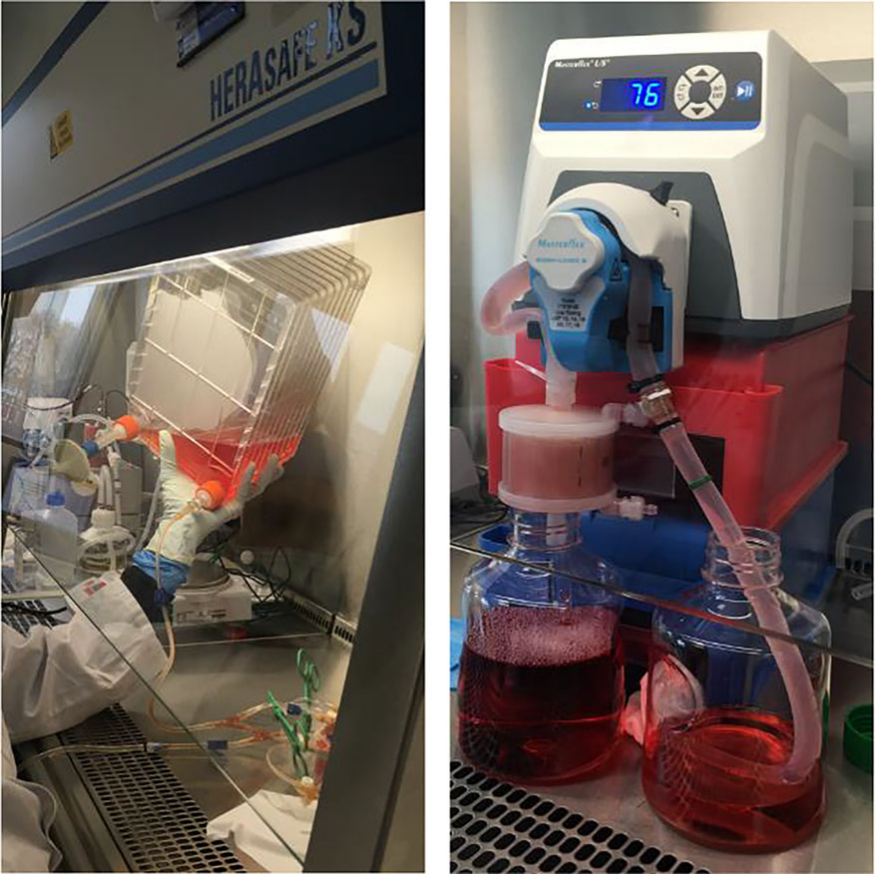 Work under microbiological safety station	GMP facility, authorised by health agencies A in B cleanrooms, environmental monitoring 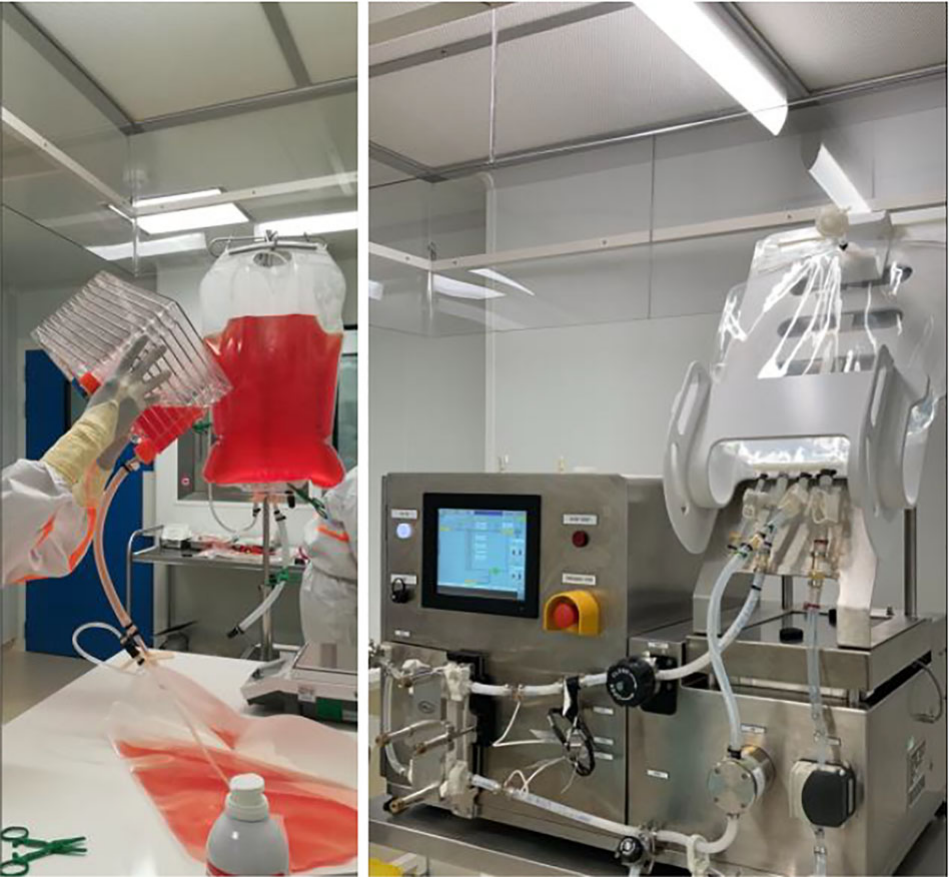 Work under FFU to maintain aseptic conditions
Manufacturing process	Process validation	Development batch	Pre‐run batch
	Vesiculation of cells	T75 culture flask	Multi‐layered culture vessels
	Culture media and reagents	Open system with bottles	Closed system with bags
	Clarification	Centrifuge	In‐line filtrations steps
	Purification and concentration by TFF	Research‐grade filters	Sterile single‐use filter compatible with TFF system
	Fill and finish	Plastic tube	Sterilising filtration and pharmaceutical glass vials
QC	Tests methods and specification	Guidelines of EV society	Accordance with EP
	Particle quantification	NTA	NTA and MACSPlex Exosome kit
	Safety tests	Not performed	In vivo toxicity and tumorigenicity studies Biological products safety tests
	Stability tests	Not performed	Accordance with ICHQ1A

Abbreviations: CoA, certificate of analysis; CoO, certificate of origin; EC, European conformity; EP, European Pharmacopeia; GMP, good manufacturing practice; ICHQ, International Council for Harmonisation Quality; NTA, nanoparticle tracking analysis; QC, quality control; RUO, research use only; TFF, tangential flow filtration.

##### 2.1.2.1 Culture and Vesiculation of CPC

Cryo‐preserved CPC were thawed and seeded onto vitronectin (Life Technologies, #A31804)—coated multi‐layered culture vessels and cultured for 5 days at 37°C, 5% CO_2_ in a humidified incubator. During the first 3 days, cells were expanded in complete, rich, medium. On the third day post‐thaw (day+3), the media was exchanged for a poor, ‘vesiculation’ medium. This minimal medium was designed by eliminating from the complete medium all components containing proteins or other complex biological molecules, that could contaminate the final product and alter its quality. It retains only the nutrients essential for CPC survival during the 2 days of vesiculation. Cells were cultured for 2 additional days in this minimal medium (up to day+5).

During this 2‐day vesiculation period, the cells produce the Cell Conditioned Medium (CCM) that includes the biologically active compounds.

##### 2.1.2.2 Clarification and Purification and Concentration of the EV‐Enriched Secretome

CCM was collected on the fifth day post‐thaw (day+5) and cleared via in‐line filtration to eliminate remaining cells, debris, larger vesicles and other cellular impurities.

The CCM was purified, concentrated and buffer‐exchanged to Dulbecco's Phosphate‐Buffered Saline 1X (PBS 1X; Corning, #21‐031‐LX) by TFF Allegro CM150 (PALL/Sartorius) using a sterile, single‐use, filter of 30 kDa cut‐off, which, on the basis of preliminary comparative studies, was found to provide the optimal trade‐off between retention of a biologically active cargo and purity of the final product.

##### 2.1.2.3 Sterilising Filtration and Fill and Finish

In accordance with the regulations governing the aseptic manufacture of biological products, the TFF retentate was subjected to sterilising filtration through a 0.2 µm‐membrane sterile capsule, aliquoted into glass vials, frozen and stored between –65°C and –85°C.

### 2.2 Characterisation of CPC During the Vesiculation Process

In‐process monitoring of cells and media was performed to ensure process control as described below and in Figure 1b.

#### 2.2.1 Number of Viable Cells and % Viability

CPC were counted to determine the number of viable cells and % of viability during the manufacturing process using a NucleoCounter NC‐200 automated cell counter with Via1‐Cassette (ChemoMetec, #941‐0012) according to EP 2.7.29 at thaw, on day+3, and on day+5.

#### 2.2.2 Identity by Flow Cytometry

Flow cytometry was performed using a MACSQuant 10 Flow Cytometer (MQ10, Miltenyi Biotec) to determine the relative expression levels of protein markers of hiPSC: SOX2 and NANOG; of CPC: CD56 and CXCR4; and of differentiated cardiomyocytes: cardiac Troponin T (cTNT) and alpha Myosin Heavy Chain (αMHC). A commercially available hiPSC line (Life Technologies) and human iCell Cardiomyocytes (FCDI, #01434) were used as control cell types. These analyses were performed according to manufacturer's instructions. Intracellular staining was performed using Inside stain kit (Miltenyi Biotec, #130‐090‐477) according to the manufacturer's instructions. Flow cytometric analyses were performed on day+3 and day+5.

#### 2.2.3 Identity by Bulk RNA Sequencing

For bulk RNA expression analysis on the CPC at day+3 and day+5, RNA was extracted from cell pellets using the Qiasymphony according to the manufacturer's directions, and sequenced on the Illumina NovaSeq 6000 platform using standard techniques. Undifferentiated hiPSC and human iCell Cardiomyocytes 2 (FCDI, #CMC‐100‐012‐001) were used as controls. Differential gene expression was determined on normalised data. A heat map was generated based on a hierarchical clustering analysis using the UPGMA clustering method, with correlation distance metric in TIBCO Spotfire software v11.2.0. The genes included in the heatmap are genes expected to be expressed at difference stages of cardiomyocyte differentiation, as well as in related, off‐target cells.

#### 2.2.4 Residual hiPSC Testing

Residual hiPSC testing was performed on CPC collected on day+3 and on day+5 (i.e., at the start and the end of the vesiculation process) using a digital droplet PCR (ddPCR) method, a proprietary technology for FCDI. Briefly, the method was developed to detect long non‐coding RNA markers of undifferentiated hiPSC cells. The Limit of Detection (LOD) for the assay was established by hiPSC spike‐in experiments.

#### 2.2.5 Conventional Karyotype

A conventional RHG‐banding karyotype analysis was performed on CPC at day+5 to evaluate genomic stability. Chromosomal testing was carried out on at least 20 cells undergoing mitosis.

#### 2.2.6 Adventitious Agent Testing

Adventitious agent testing was performed at the end of CPC culture at day+5 (F‐PERT assay for the detection of reverse transcriptase activity; transmission electron microscopy for bulk product; detection of virus contaminant using Vero, BT & ST cell lines). Viral safety evaluations were in accordance with nternational Council for Harmonisation Quality (ICHQ) guidelines (Q5A ‘*Viral safety evaluation of biotechnology products derives from cell lines of human or animal origin*’ (EMA 2018) and Q5D ‘*Derivation and characterization of cell substrates used for production of biotechnological/biological products’* (EMA 2018)) and were outsourced to Texcell SA, Évry‐Courcouronnes, France (https://texcell.com/).

### 2.3 Characterisation of the EV‐Enriched Secretome

#### 2.3.1 Nanoparticle Tracking Analysis

To determine the size (mean and mode) and number of particles in media throughout the manufacturing process, samples at various stages were collected and analysed by Nanoparticle Tracking Analysis (NTA) using a NanoSight LM10 instrument (Malvern Panalytical), equipped with a 405‐nm laser. Data are an average of three technical replicates, each with five videos of 1 min each, with the instrument maintained at 27°C. Data were analysed with the NTA 3.2 software. A camera level of 15 and a detection threshold of 4 were used for these analyses.

#### 2.3.2 Particle Identity and Quantification of Particles

*Particle identity*: To characterise the surface markers of particles in the EV‐enriched secretome preparations, the MACSPlex Exosome Kit (Miltenyi Biotec, #130‐108‐813) was used following the manufacturer's instructions. In this kit, 37 surface epitopes and two isotype controls can be detected. Samples were incubated overnight with the antibody‐coated MACSPlex Exosome Capture Beads (comprising 39 different bead populations distinguishable by their unique combinations of FITC and PE fluorescence intensities). Then, samples were incubated 1 h with MACSPlex Exosome Detection Reagent (a blend of anti‐CD9, anti‐CD63 and anti‐CD81 antibodies). Sandwich complexes were formed between Capture Bead, the captured particles and the Exosome Detection antibodies. Complexes were analysed using a MQ10 Flow Cytometer (Miltenyi Biotec).

*Quantification of particles*: With the understanding that bead‐based flow cytometry of EV can be semi‐quantitative (Wiklander et al. 2018), a correlation between NTA concentration (in particles/mL) and Median Fluorescence Intensity (MFI) of CD9 expression using MACSPlex Exosome Kit was established (*manuscript in preparation*). Briefly, samples at various stages of secretome purification were analysed by both NTA and by MACSPlex Exosome Kit. Multiple samples and/or individual sample dilutions of EV‐enriched secretome (*n* = 120) were analysed by NTA and for CD9 MFI. The concentration of particles, as measured by the NTA, was found to be correlated to the CD9 MFI for samples (*data not shown*). A correlation coefficient could thus be established. This method was employed to quantify particles at various stages of our process. For each test sample, the quantification of particles was calculated using the established correlation coefficient.

#### 2.3.3 Cryo‐Electron Microscopy Analysis

Cryo‐Electron Microscopy (Cryo‐EM) studies were performed using standard methods at the UW‐Madison Cryo‐Electron Microscopy Research Center (Madison, WI, USA). Briefly, samples were spotted onto mesh grids (Quantifoil R 1.2/1.3 Cu 200) that had previously been glow discharged (60 s at 20 mA with the GloQube). Multiple applications (3 µL per drop) onto the grids were utilised. Loaded grids were plunge frozen using a ThermoFisher Mark IV Vitrobot (Vitrobot conditions: 4°C, 95% humidity, 0.5 s drain time, 30 s wait time for all grids). Images were collected on the Talos Arctica at 200 kV using a Gatan K3 direct electron detector in counting mode with an energy filter at a 20 eV slit width. For high‐resolution imaging, the following magnification was set: 79kx, spot size 4, C2 aperture 70, C2 lens power 40.939%, objective aperture 100, pixel size 1.1Å, dose 48.0 e‐/Å2, exposure time 4.2 s, defocus‐2 m.

#### 2.3.4 ONi Super‐Resolution Nanoparticle Imaging

Single particle analysis was performed by ONi Nanoimager. The ONi is a d‐STORM capable super‐resolution fluorescence microscope. Samples were assayed using the EV Profiler Kit (Oxford Nanoimaging, #210917‐01) according to the manufacturer's directions. The principle of this kit is that objects are immobilised onto a chip by affinity capture and then are probed for tetraspanin marker expression. The ONi software identifies clusters of fluorescence, categorises the cluster sub‐type, and then determines the percentage of each cluster sub‐type. Two clusters are of the same sub‐type if they express the same combination of tetraspanin markers. For example, clusters defined as the CD9 single positive sub‐type are positive for CD9 and negative for CD63 and CD81, whereas the clusters defined as the CD9 + CD63 double positive sub‐type are positive for CD9, positive for CD63 and negative for CD81. Chips were imaged on the ONi microscope using 488, 560 and 640 lasers. The instrument temperature was set to 30°C, an illumination angle of 52° was used, and 2500 frames were captured for each sample.

#### 2.3.5 Residual DNA

Quantification and characterisation of residual DNA was performed under method A of EP 2.6.35. Briefly, two ALU consensus sequences were selected as targets. These are repetitive sequences ubiquitous in human cells (Weisenberger et al. 2005). The evaluation and quantification of these targets enabled the quantification of DNA fragments above the recommended 200 base pair (bp) in length.

#### 2.3.6 EV‐RNA Transcriptomics

RNA was extracted from EV‐enriched secretome preparations using the Wako microRNA Extractor SP kit (Wako, #295‐71701) following the manufacturer's instructions. Quantity and quality of RNA were determined using a 2100 Bioanalyser with the Eukaryote Total RNA Pico Assay (Agilent technologies). A short read sequencing library was prepared using QIAseq miRNA Library which was QC tested using the Agilent Bioanalyser HS DN chip (Agilent Technologies). The cDNA was purified using QMN beads (Qiagen). Sequencing was performed using NovaSeq6000 on the Illumina NGS Systems. For bioinformatic analysis of sequencing data, FastQC v0.11.9 was used to determine the quality of the raw reads. Trimmomatic v0.39 was used to trim the adaptors from the raw reads. The trimmed reads were used for read mapping, and quantification was performed using miRge3.0 pipeline implemented in python with default settings (miRge 3.0 uses Bowtie v1.3.0 and SAMtools v1.7 for read mapping and quantification). miRbase22 was used for miRNA annotations.

#### 2.3.7 Protein Concentration

Protein concentration in the test samples was determined using BC Assay (Bicinchoninic acid method) kit (UPTIMA/Interchim, #UP408404A) according to the manufacturer's instructions under method 4 of EP 2.5.33. Colorimetric reading was performed on a spectrophotometer (Multiskan Sky Spectrophotometer, Thermo Scientific).

#### 2.3.8 Proteomics

The protein content of the EV‐enriched secretome was determined by mass spectrometry using standard methods. Briefly, the protein mixture was reduced, alkylated and digested prior to LC‐MS/MS analysis. Online chromatography was performed with an RSLCnano system (Ultimate 3000, Thermo Scientific) coupled to an Exploris 480 with a Nanospay Flex ion source (Thermo Scientific). Peptides were first trapped on a C18 column (75 µm inner diameter × 2 cm; nanoViper Acclaim PepMap 100, Thermo Scientific) with buffer A (2/98 MeCN/H2O in 0.1% formic acid) at a flow rate of 2.5 µL/min over 4 min. Separation was then performed on a 50 cm × 75 µm C18 column (nanoViper Acclaim PepMap RSLC, 2 µm, 100Å, Thermo Scientific) regulated to a temperature of 50°C with a linear gradient of 2%–30% buffer B (100% MeCN in 0.1% formic acid) at a flow rate of 300 nL/min over 91 min. MS full scans were performed in the ultrahigh‐field Orbitrap mass analyser in ranges m/z 375–1500 with a resolution of 120,000 at m/z 200. Protein molar and mass percentages were estimated by using Top 3 (Silva et al. 2006) (including proteins with less than three peptides) as the Protein Quantification Index and the direct proportionality model (Ahrné et al. 2013). The experiments were performed in five replicates at the *Institut Curie, Université Paris Sciences & Lettres (PSL), Centre de Recherche, CurieCoreTech Spectrométrie de Masse Protéomique*, Paris, France.

#### 2.3.9 Host‐Cell Protein Assessment

To determine process‐related impurities derived from the mother cell, Glucose‐regulated protein 94 (GRP94) assays were performed by ELISA using ImmunoSet Grp94 ELISA development set (Enzo Life Sciences, #ADI‐960‐077) for the quantitative determination of GRP94 protein content in samples before and after TFF. GRP94 is a protein in the lumen of the endoplasmic reticulum present only in eukaryotic cells. Identification of this protein by immunoblotting is commonly used to describe the purity of the final product (Dozio and Sanchez 2017; Djeungoue Petga et al. 2024). This assay was used for host‐cell (mother cell) protein assessment to quantify non‐secretome associated protein levels. This assay was performed following the manufacturer's instructions. Colorimetric reading was performed on a spectrophotometer (Multiskan Sky Spectrophotometer, Thermo Scientific).

#### 2.3.10 Pharmaceutical Technical Procedures and Physicochemical Methods

Testing has been made according to EP requirement for parenteral preparations. The extractable volume was performed by withdrawing the volume contained in the glass vials according to EP 2.9.17. The unit uniformity dosage was performed by quantifying particle concentration according to EP 2.9.6. The clarity and degree of opalescence of liquids and the degree of coloration of liquids were assessed by visual examination of the glass tubes according to EP 2.2.1 and EP 2.2.2. The integrity of the glass tubes was checked using a vacuum chamber according to EP 5.1.1. The pH of liquid solution was determined using a pH‐meter under EP 2.2.3.

#### 2.3.11 In Vitro Functional Assays

##### 2.3.11.1 Proliferation Assay (Potency Assay)

The functionality of EV‐enriched secretome preparations was evaluated using a novel and proprietary in vitro potency assay based on the proliferation of Human Umbilical cord Vein Endothelial Cells (HUVEC). HUVEC proliferation was quantified by measuring bromodeoxyuridine (BrdU) incorporation as follows. Briefly, HUVEC were purchased from Lonza (#C2519A), expanded, and cryopreserved according to the manufacturer's instructions. Cryo‐preserved vials of the HUVEC cell bank were thawed and plated onto round‐bottomed 96‐well‐plates (Dutscher, #353077) at 20,000 cells/well in complete media (EBM‐2 media (Lonza, #CC‐3156), supplemented with EGM‐2MV (Lonza, #CC‐4147)) and incubated for 24 h at 37°C, 5% CO_2_ in a humidified incubator. Media were exchanged to complete media (positive control), poor media (EBM‐2 media alone; negative control), poor media with final product (test conditions, 50 µL = 9.75 × 10^9^ particles and 40 µg of protein) or poor media with PBS 1X (vehicle control). BrdU (Sigma, #11647229001) was added to all conditions, according to the manufacturer's directions, and HUVEC were cultured a further 24 h under the testing conditions. BrdU was detected within cells using an anti‐BrdU antibody and a colorimetric assay according to the manufacturer's directions. Signal was detected by spectrophotometer (Multiskan Sky Spectrophotometer, Thermo Scientific). A relative HUVEC proliferation rate is determined by comparing the quantity of BrdU in positive controls, negative controls and test conditions. An increase in BrdU signal from test conditions over the negative control indicates that the test condition stimulated HUVEC proliferation. Values were double normalised (baseline [negative control] subtracted, and normalised to the positive control).

##### 2.3.11.2 Cardiomyocyte Survival Assay

A novel cardiomyocyte survival assay was developed in which human cardiomyocytes are stressed with staurosporine (FCDI proprietary technology). Briefly, iCell Cardiomyocytes 2 (FCDI, #CMC‐100‐012‐001) were plated at 50,000 cells/well of a fibronectin‐coated 96‐well plate. Cells were cultured in iCell Cardiomyocyte Maintenance Medium (iCMM, FCDI, #M1003) for 4 h at 37°C, 5% CO_2_ in a humidified incubator. The media was then refreshed 4 h post plating with a full media exchange (again with iCMM). Cells were cultured for up to 7 days, with full media exchanges every 2–3 days. After a minimum of 4 days, cells were pre‐treated with iCMM with NucSpot Live 650 dye (Biotium, #40082) (no‐stress condition); or to iCMM with NucSpot Live 650 dye, and staurosporine (Abcam, #ab146588) (stress condition). Dye, PBS 1X, DMSO concentrations, and final well volumes were equivalent in all wells, throughout the assay. After this pre‐incubation, the pre‐treatment media were removed, and the wells were rinsed with fresh iCMM. No‐stress cells were then fed with iCMM with NucSpot Live 650 dye and PBS 1X (positive control). In parallel, stressed cells were fed with iCMM with NucSpot Live 650 dye supplemented with PBS 1X alone (negative control), or with increasing concentrations of test material (test conditions, 1× = 5.79 µL = 1.13 × 10^9^ particles and 4.63 µg protein), maintaining PBS 1X final volumes the same for all conditions. Wells were imaged on an Incucyte every hour for 24 h, and nuclei counts were determined. Values were double normalised (baseline [negative control] subtracted, and normalised to the positive control).

##### 2.3.11.3 Scratch Wound Healing Assay

A HUVEC scratch wound healing assay (developed by Essen BioSciences for the Incucyte) was performed according to the manufacturer's instructions. Briefly, HUVEC were purchased from PromoCell (#c‐12200), expanded and cryo‐preserved according to the manufacturer's instructions. Cryo‐preserved vials of the HUVEC cell bank were thawed and plated onto ImageLock 96‐well plates (Essen BioSciences, #210102) at 10,000 cells/well in complete media (Endothelial Cell Basal media supplemented with the Endothelial Cell Growth Medium Supplement Pack (PromoCell)) and incubated for 48 h at 37°C, 5% CO_2_ in a humidified incubator. The monolayer of cells was scratched using a Wound Maker (Essen BioSciences, #4493). Media were exchanged to complete media (positive control), poor media (Endothelial Cell Basal media alone; negative control) or poor media with increasing doses of the final product (test conditions, 1× = 5.79 µL = 1.13 × 10^9^ particles and 4.63 µg protein). Using an Incucyte with the Scratch Wound Healing Module, plates were imaged every 3 h for a total of 18 hs. Wound closure was determined using the manufacturer's software, and values were double normalised (baseline [negative control] subtracted, and normalised to the positive control).

#### 2.3.12 In Vitro Alloreactivity Assays

The intended use of the EV‐enriched secretome IMP is for the allogenic treatment of heart failure patients. To determine the level of alloreactivity of the final product, the alloreactive activations of human Natural Killer (NK) and human PBMC were tested as follows.

*NK cells degranulation*: Human NK cells were isolated from human PBMC (healthy donors from *Etablissement Français du Sang (EFS)*) by negative selection using immune‐magnetic cell sorting (Miltenyi Biotec, #130‐092‐657) according to the manufacturer's instructions and as described elsewhere (Lima Correa et al. 2021). Experiments were conducted with human NK cells primed overnight with recombinant human interleukine‐15 (IL‐15) (50 ng/mL) (Sigma) in RPMI‐1640 complete medium supplemented with 10% Foetal Bovine Serum (FBS) to ensure their proper expression of human NK cells activating receptors and functionality. Cytokine‐activated human NK cells were cultured in the presence of human K562 cells (ATCC, #CCL‐243; positive control), or PMA/ionomycin (Beckman Coulter, #C11102; positive control), PBS 1X (50 µL; vehicle control) or final product (50 µL); and labelled with an anti‐CD107a‐APC antibody (Miltenyi Biotec, #130‐111‐847). After 18 h, cells were harvested, washed and stained with CD16‐PE, CD56‐PE, CD8‐APC Vio770, CD4 Viogreen specific antibodies and 7‐AAD (Miltenyi Biotec), and the expression of CD107a was analysed on CD16^+^ CD56^+^ human NK cells using a MQ10 Flow Cytometer (Miltenyi Biotec).

*PBMC alloreactive activation*: Human PBMC were purified from blood samples from healthy donors (from *EFS)* using Ficoll gradient (Eurobio Scientific, #CMSMSL01) centrifugation and freshly used. Human PBMC were cultured alone or in the presence of PMA/ionomycin 1X (Beckman Coulter, #C11102; positive control), PBS 1X (50 µL; vehicle control) or final product (50 µL). After 4 h, supernatants were harvested for cytokine measurements. A bead‐based multiplex immunoassay, MACSPlex Cytotoxic T/NK Cell Kit (Miltenyi Biotec, #130‐125‐800), was used to measure the levels of multiple cytokines, including Interferon‐gamma (IFN‐γ) and Interleukine‐2 (IL‐2). Concentrations were calculated using Express mode acquisition on MQ10 Flow Cytometer (Miltenyi Biotec).

#### 2.3.13 Toxicity and Tumorigenicity Studies

*Animal welfare*: These studies were completed in accordance with the Directive 2010/63/UE of the European parliament and of the Council of 22 September 2010 for the protection of animals used for scientific purposes.

*GLP compliance*: In vivo studies were executed to explore the safety of the final product: two acute toxicity studies (one in rats and one in mice, both immunocompetent) and one tumorigenicity study in nude mice (immunodeficient). These regulatory studies were performed in compliance with GLP regulations at European Research Biology Center (ERBC; Certificate of compliance n°2021/17 with GLP according to directives 2004/9/CE and 2004/10/CE; https://www.erbc‐group.com/). For these GLP studies, the IMP dose chosen was set to 10‐fold higher particles/kg than the highest dose intended in the human clinical trial. This 10‐fold increase is to ensure a significant safety margin.

*Toxicity studies*: Rats used were Specific Pathogen Free (SPF) Sprague Dawley. Mice were SPF BALB/c. Three doses of the final product or PBS 1X (vehicle control) were administered intravenously (IV). Animals were followed clinically for 14 days and then sacrificed and analysed.

*Tumorigenicity study*: Mice used were Specific and Opportunistic Pathogen Free Nude: BALB/c Nude J (nu/nu). A single dose of the final product, PBS 1X (vehicle control) or HeLa cells (positive control) was administered by Sub Cutaneous (SC) injection. Animals were followed clinically for 91 days and then sacrificed and analysed.

### 2.4 Stability Measurements

The stability of the product has been assessed in accordance with ICHQ 1A ‘*Stability Testing of New Drug Substances and Products*’ (EMA 2018). Stability of the final product was determined using the following parameters: quantification of particles by MACSPlex Exosome Kit, size of particles by NTA, expression of CD9, CD63 and CD81 by MACSPlex Exosome Kit, product potency in the HUVEC proliferation assay and sterility. Stability tests were conducted approximately every 3–6 months of storage at temperatures between –65°C and –85°C.

### 2.5 Safety Tests

Microbiological sterility was assessed by direct inoculation according to EP 2.6.1 and 2.6.27. Mycoplasma testing was performed by qPCR in accordance with EP 2.6.7. Endotoxin testing was performed according to EP 2.6.14 Method D (Kinetic chromogenic LAL).

## 3 Results

*Summary of manufacturing process and QC release method development*: Previously, we had established that RUO EV‐enriched secretome preparations derived from RUO hiPSC‐derived CPC had in vitro, pro‐angiogenic and anti‐inflammatory effects (Lima Correa et al. 2021), and they improved cardiac function in animal models of chronic heart failure (Kervadec et al. 2016; El Harane et al. 2018). Several years of work were therefore undertaken to adapt the hiPSC to CPC differentiation, CPC vesiculation, and EV‐enriched secretome isolation processes to GMP‐ready, scalable, manufacturable processes. CPC viability, identity, purity, stability through cryo‐preservation and vesiculation potential were key metrics for the CPC differentiation process development. For the development of the CPC vesiculation process, critical process parameters included: CPC post‐thaw‐viability, plateability and expandability, as well as secretome potency. For the development of the secretome isolation process, critical product quality parameters included: EV‐enriched secretome in vitro potency, yield and manufacturability. Prototype products were tested in animal models at key product development time points. An early prototype was found to be therapeutically effective in a mouse model of post‐ischemic chronic heart failure (*manuscript in preparation*) and a late prototype was found to be therapeutically effective in a non‐ischemic, doxorubicin‐induced cardiac insufficiency rat model (Desgres et al. 2023).

Final process development, optimisation and lock‐down have resulted in the proprietary manufacturing process illustrated in Figure 1, suitable for Phase I clinical testing. The manufacturing process comprises: 1/hiPSC to CPC differentiation and cryo‐preservation, optimised to maintain CPC viability and expandability after thaw; 2/CPC thaw and plating, optimised for CPC viability, plateability and survival; 3/CPC culture in rich but serum‐free CPC culture media to maximise yield and minimise further differentiation; 4/CPC vesiculation in minimal (poor) media to minimise media component contamination of the spent media; 5/clarification of spent media using in‐line filtration to remove large debris and large EV and to minimise TFF filter clogging; 6/isolation, concentration and buffer exchange of the EV‐enriched secretome using a 30 kDa cut‐off TFF method (the size cut‐off was determined experimentally so as to maximise in vitro potency and product yield, *data not shown*); 7/sterilizing filtration and glass‐vial filling; 8/storage between –65°C and –85°C.

Some of the major differences between the RUO process and the optimised GMP‐compatible and scalable processes are summarised in Table 1.

Throughout manufacturing process optimisation, relevant in‐process monitoring, product characterisation and product release testing strategies were developed and optimised to ensure process control, robustness, product efficacy and patient safety. Process/product understanding, combined with a careful combination of regulatory guidance for several separate classes of drugs led to our final panel of in‐process testing and QC release tests (Tables 2 and 3). This was necessary but challenging due to a lack of clear guidance for EV‐based therapeutics at this very early stage of the field.

**TABLE 2:** Quality controls of CPC during the vesiculation. Parameter, method, specifications, results.

Parameter	Method	Specifications	Results	
			Day+3	Day+5
Number of viable cells	NucleoCounter NC‐200 (DAPI/AO staining) (EP 2.7.29)	Informative	9.5 × 10^9^	8.1 × 10^9^
% viability		>70	91.5	91.5
Microbiological sterility	Bact/Alert (EP 2.6.1 and 2.6.27)	Negative at 10 days	Negative at 10 days	Negative et 10 days
Mycoplasma	qPCR (EP 2.6.7)	<10 CFU/mL	<10 CFU/mL	<10 CFU/mL
Endotoxin	Kinetic chromogenic LAL (EP 2.6.14)	<2 EU/mL	<2 EU/mL	<2 EU/mL
Karyotype	RHG‐banding	No abnormalities	/	No abnormalities
Adventitious agent testing	F‐PERT assay for the detection of reverse transcriptase activity (ICHQ5A, ICHQ5D) (EP 5.2.3 and 5.2.12)	No reverse transcriptase activity	/	No reverse transcriptase activity
	Transmission electron microscopy for bulk product (quantitative) (ICHQ5A) (EP 5.2.3 and 5.2.12)	No virus observed	/	No virus observed
	Detection of virus contaminant using Vero, BT & ST cell lines (ICHQ5A) (EP 5.2.3 and 5.2.12)	No viral contaminant detected	/	No viral contaminant detected
Identity	Flow cytometry (EP 2.7.24)	<5% SOX2/NANOG >95% CD56/CXCR4	<5% SOX2/NANOG >95% CD56/CXCR4	<5% SOX2/NANOG >95% CD56/CXCR4
		cTNT MFI: increasing	6.3	12.3
		αMHC MFI: increasing	37.7	46.3
Residual hiPSC testing	ddPCR	<0.37 copies/µL (corresponding to <2% of residual hiPSC)	Below detectable limits (i.e., not detectable)	Below detectable limits (i.e., not detectable)

Abbreviations: αMHC, alpha myosin heavy chain; AO, acridine orange; BT, bovine turbinate; CFU, colony forming unit; cTNT, cardiac troponin T; DAPI, 4′,6‐diamidino‐2‐phenylindole; ddPCR, digital droplet PCR; EP, European Pharmacopeia; EU, endotoxin unit; hiPSC, human induced pluripotent stem cell; ICHQ, International Council for Harmonisation Quality; LAL, limulus amebocyte lysate; MFI, median fluorescence intensity; qPCR, quantitative real‐time PCR; ST, swine testicular.

**TABLE 3:** Quality controls of the final product. Parameter, method, specifications, results.

Parameter	Method	Specifications	Results
Particle concentration	NTA	Informative	195 × 10^9^ particles/mL
Particle size distribution		Between 50 and 200 nm	Mean: 134 nm Mode: 95 nm
Particle identity	Flow cytometry tetraspanins: CD9/CD63/CD81 by MACSPlex (Miltenyi) (EP 2.7.24)	MFI CD9 > 10 MFI CD63 > 10 MFI CD81 > 10	MFI CD9 = 27 MFI CD63 = 132 MFI CD81 = 140
Particle quantification	Correlation between NTA and MACSPlex results	>125 × 10^9^ particles/mL	196 × 10^9^ particles/mL
Residual DNA, quantification	qPCR (EP 2.6.35)	Informative	>80 bp fragments = 2.2 µg/mL >200 bp fragments = 1.3 µg/mL <200 bp fragments = 0.9 µg/mL
Protein concentration	BCA assay (EP 2.5.33; method 4)	Informative	0.8 mg/mL
Extractable volume	EP 2.9.17	2 mL +/– 5%	1.96 mL
Uniformity by MACSPlex, MFI CD9	EP 2.9.6	85%–115% of the mean value	90%–100%
Microbiological sterility	Bact/Alert (EP 2.6.1 and 2.6.27)	Negative at 10 days	Negative at 10 days
Mycoplasma	qPCR (EP 2.6.7)	<10 CFU/mL	<10 CFU/mL
Endotoxin	Kinetic chromogenic LAL (EP 2.6.14)	<2 EU/mL	<2 EU/mL
Appearance	Clarity and degree of opalescence of liquids; and visible particles (EP 2.2.1 and 2.9.20)	Clear and no visible particles	Clear and no visible particles
	Degree of coloration of liquids (EP 2.2.2)	Colourless	Colourless
Integrity	EP 5.1.1 and USP 1207	Package integrity	Package integrity
Host cell protein assessment	GRP94 assays	Informative	122 ng/mL
pH	Potentiometric determination of pH (EP 2.2.3)	7.20–7.50	7.48
Potency in vitro	Proliferation assay	Increased proliferation > 20%	47%
Immunogenicity in vitro	Degranulation of human NK cells (ICHS8)	Lack of % of expression of CD107a compared with the control	Lack of % of expression of CD107a compared with the control
	Activation of allogeneic human PBMC assessed by secretion of INF‐γ and IL‐2 (ICHS8)	Lack of increased secretion of INF‐γ and IL‐2 compared with the control	Lack of increased secretion of INF‐γ and IL‐2 compared with the control
In vivo GLP studies	Toxicity study in BALB/c mice and Sprague Dawley rat (ICHS3A)	No safety concerns	No safety concerns
	Tumorigenicity study in BALB/c Nude J mice (EP 5.2.3)	No safety concerns	No safety concerns

Abbreviations: BCA, bicinchoninic acid; bp, basepair; CFU, colony forming unit; EP, European Pharmacopoeia; EU, endotoxin unit; GLP, good laboratory practice; ICHS, International Council for Harmonisation Safety; IL‐2, interleukin‐2; INF‐γ, interferon‐gamma; LAL, limulus amebocyte lysate; MFI, median fluorescence intensity; NK, natural killer; NTA, nanoparticle tracking analysis; PBMC, peripheral blood mononuclear cells; qPCR, quantitative real‐time PCR; USP, United States Pharmacopeia.

Hereafter are detailed the results of in‐process testing at each step of the manufacturing process, product QC release testing, GLP animal safety studies and stability monitoring for a batch of FCDI CTC1‐EV‐enriched secretome, fully produced in compliance with GMP and at Phase I clinical manufacturing scale.

### 3.1 GMP‐Compliant Process for the Manufacturing of EV‐Enriched Secretome From CPC

All GMP compatible process (including documentation, raw materials, equipment, human resources, facility, manufacturing process and QC) are based on a risk‐based approach in accordance with ICHQ9 ‘*Quality risk management*’ (ICH Guideline Q9 on Quality Risk Management).

The entire GMP large‐scale process for the manufacturing of an EV‐enriched secretome is summarised in Figure 1a. The three main steps include: 1/vesiculation of CPC; 2/clarification and purification and concentration of the EV‐enriched secretome; 3/sterilizing filtration and fill and finish. All processes were conducted under aseptic conditions in cleanrooms according to GMP regulation to maintain aseptic conditions.

Cryo‐preserved CPC were cultured in multi‐layered vessels. After 2 days in poor medium, spent media that contains EV were collected in bags. CCM was clarified via in‐line filtration before being purified and concentrated by TFF using a sterile filter cassette with of 30 kDa size cut‐off. The entire process resulted in ∼50‐fold concentration of EV. The TFF retentate was subjected to sterilising filtration through 0.2 µm‐membranes, resulting in a final volume of 530 mL. This final product was then aliquoted into glass vials and stored between –65°C and –85°C. A QC strategy was implemented to characterise the cells during the manufacturing process and the final product (Figure 1b).

### 3.2 Characterisation of CPC During the Vesiculation Process

**FIGURE 2:**
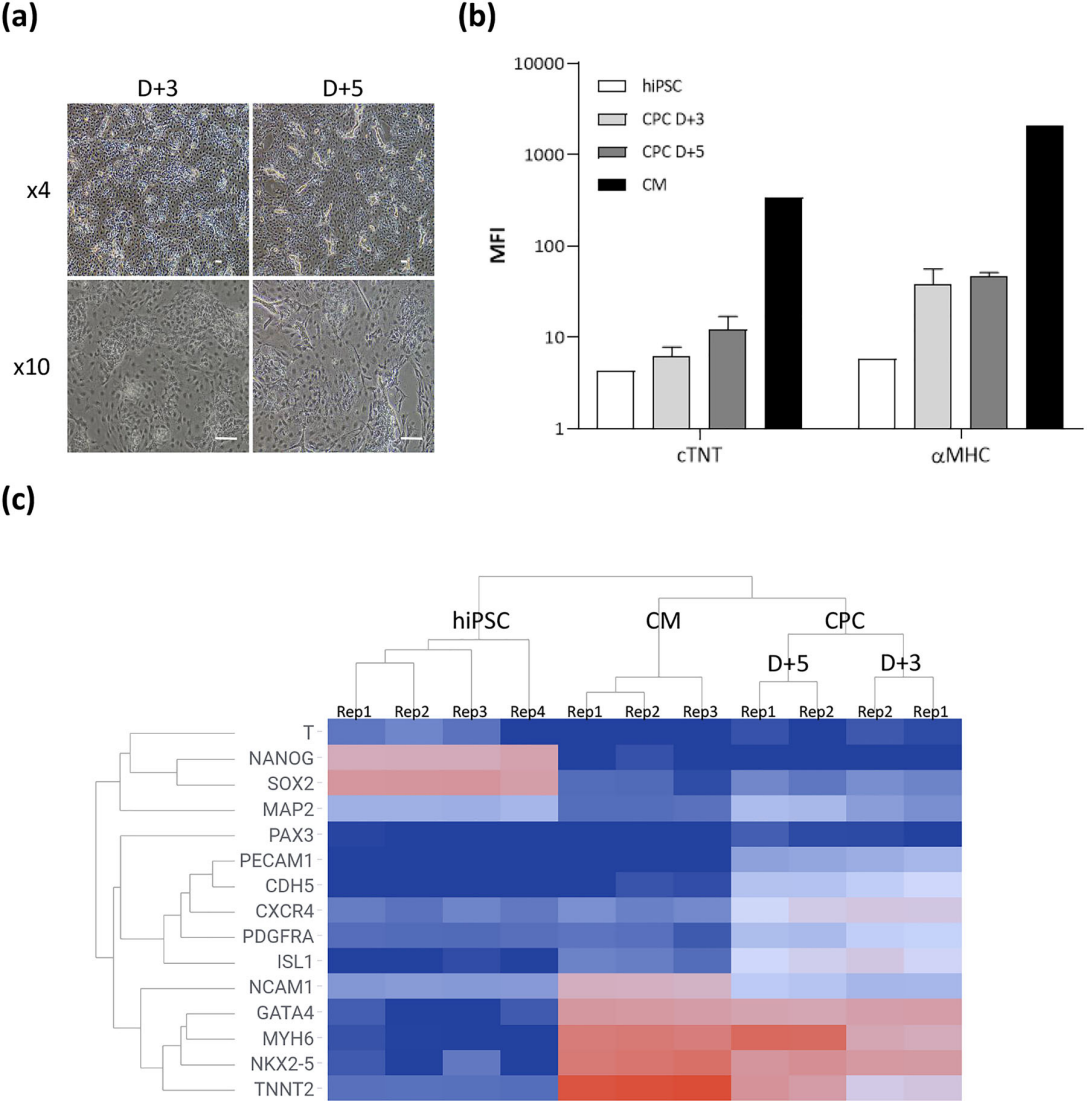
Characterisation of CPC during the vesiculation process. (a) CPC morphological analysis by microscopy at day+3 (D+3) and day+5 (D+5) of the vesiculation process. Scale bar = 100 µm. (b) Cell identity by flow cytometry. Cell marker expression profile of CPC at day+3 (D+3) and day+5 (D+5), and of hiPSC and human cardiomyocytes (CM) as controls. MFI are shown for two cardiac specific markers: cTNT (cardiac troponin T) and *α*MHC (alpha Myosin Heavy Chain). (c) Cell identity by bulk RNA sequencing. Total RNA was sequenced on the Illumina NovaSeq 6000 platform. Results of the transcriptomic analysis for two batches of CPC at the start of vesiculation at day+3 (D+3) and after vesiculation at day+5 (D+5) are compared to hiPSC and CM transcriptomes. Hierarchical clustering analysis was performed, using the UPGMA clustering method, with correlation distance metric in TIBCO Spotfire software v11.2.0. Results are represented as a heatmap. T, brachyury; NANOG, Nanog Homeobox; SOX2, SRY‐box transcription factor 2; MAP2, microtubule associated protein 2; PAX3, paired box 3; PECAM1, platelet and endothelial cell adhesion molecule 1; CDH5, cadherin 5; CXCR4, C‐X‐C motif chemokine receptor 4; PDGFRA, platelet derived growth factor receptor alpha; ISL1, insulin gene enhancer protein ISL‐1; NCAM1, neural cell adhesion molecule 1; GATA4, GATA binding protein 4; MYH6, myosin heavy chain 6; NKX2‐5, NK2 homeobox 5; TNNT2, troponin T2, cardiac type; Rep, replicate.

CPC were evaluated for quantity, safety, purity and identity before and after vesiculation (Figure 1b). The results of QC testing met all acceptance criteria, and are summarised in Table 2 and in Figure 2.

*In‐process CPC identity monitoring—morphology*: Additional in‐process characterisation of CPC were performed to confirm cell‐type morphology by microscopy (Figure 2a). CPC at day+3 and day+5 were deemed morphologically acceptable compared with the morphologies observed in previous batches.

*In‐process CPC identity monitoring—protein markers*: Specific cell surface and intracellular markers of CPC were verified by flow cytometry (Table 2; Figure 2b). The specifications were: positive expression of CPC markers NCAM/CD56 (Evseenko et al. 2010; Romagnuolo et al. 2019) and CXCR4 (Bondue et al. 2011); absence of pluripotent markers SOX2 and NANOG (Sullivan et al. 2018); and some expression of cardiomyocyte markers cTNT (Kattman et al. 2011; Romagnuolo et al. 2019) and αMHC (Cho et al. 2021), which are expected to be at higher levels than in hiPSC but not as high as in human cardiomyocyte controls. CPC at day+3 and day+5 expressed more cTNT and αMHC than hiPSC, but less than human cardiomyocyte controls, meeting specifications and confirming that CPC are on the cardiac differentiation path, are no longer hiPSC, but are not yet human mature cardiomyocytes.

*In‐process CPC identity monitoring—transcriptomics*: The identity of CPC during the manufacturing process was further verified by bulk RNA sequencing (Figure 2c). As expected, CPC did not express pluripotent markers NANOG or SOX2 (Sullivan et al. 2018) but were positive for CPC‐specific markers such as CXCR4 and PDGFRA (Bondue et al. 2011; Kattman et al. 2011; Romagnuolo et al. 2019), ISL1 (Burridge et al. 2012), NCAM/CD56 (Evseenko et al. 2010; Romagnuolo et al. 2019), GATA4 and NKX2‐5 (Burridge et al. 2012). CPC expressed less MYH6 and TNNT2 than human cardiomyocyte controls (Chen et al. 2015) and little to no off‐target markers MAP2 (neuronal marker; Soltani et al. 2005), PAX3 (skeletal muscle cell marker; Magli et al. 2019), PECAM1 (endothelial and blood cell marker; Baumann et al. 2004) or CDH5 (endothelial cell marker; Larson et al. 2004). Consistent with flow cytometry results, RNA sequencing results show that CPC at day+3 and day+5 are more mature than hiPSC but less mature than human cardiomyocyte controls. The presence of hiPSCs was not detectable (Table 2). All these data thus confirmed that the vesiculating cells retained the characteristics of CPC throughout the vesiculation process.

### 3.3 Characterisation of the EV‐Enriched Secretome

A QC strategy was developed that aims to ensure quantity, safety, purity and identity of the final product. The results of QC testing of the EV‐enriched secretome are described below and summarised in Figure 1b.

**FIGURE 3:**
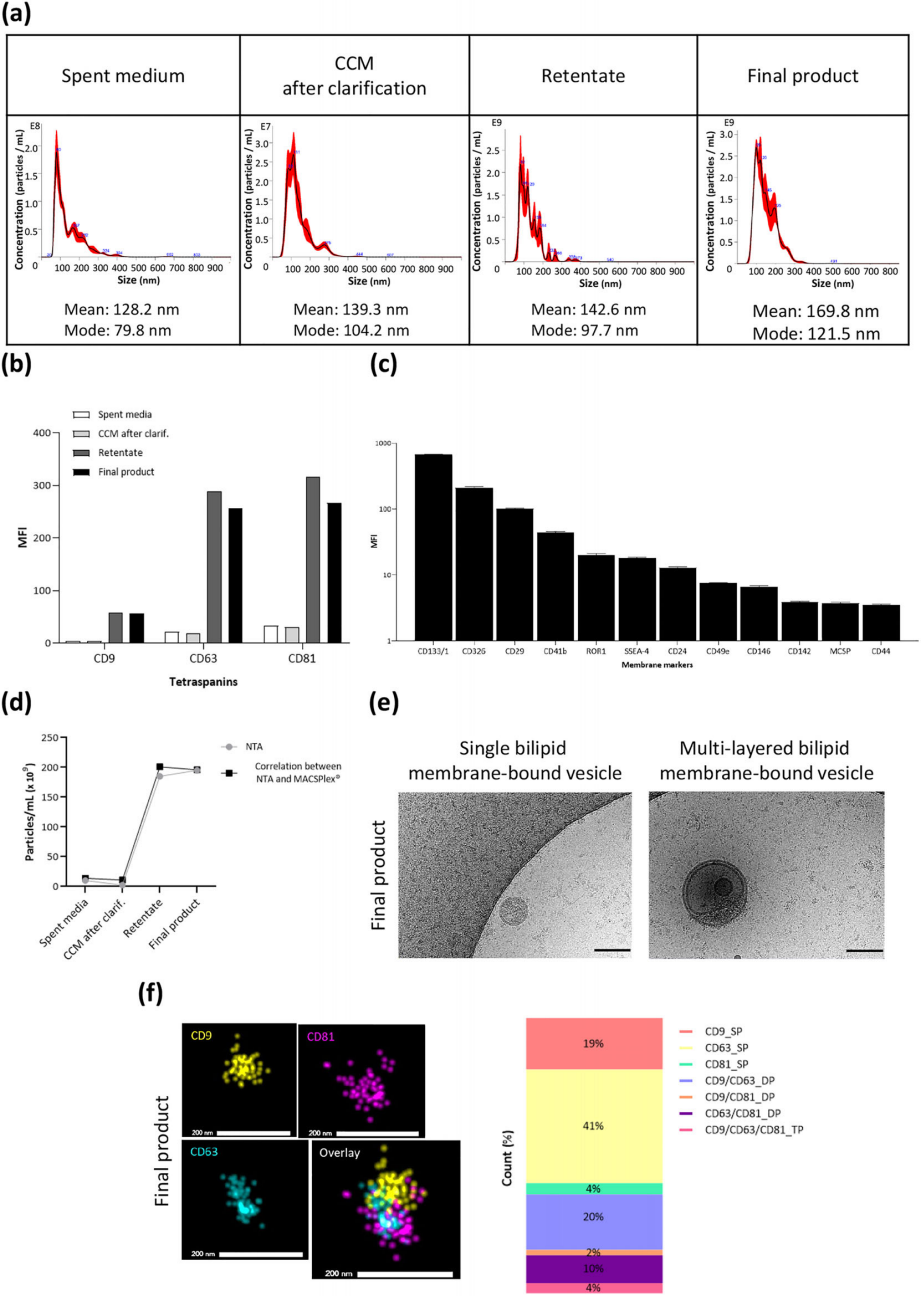
EV enrichment in the final product after purification and concentration by TFF. (a) Particle size distribution. Size distribution profiles were obtained by NTA in spent medium, in CCM after clarification, in the retentate, and in the final product. Data were analysed with the NTA 3.2 software, and the mean and mode of each condition were determined. The data presented are from a single representative experiment. (b) Particle identity and tetraspanin enrichment over the manufacturing process. MFI of CD9, CD63 and CD81 obtained by MACSPlex Exosome kit are presented for spent media, CCM after clarification, retentate and final product. (c) Particle surface marker expression. An abundance of surface proteins on EV in the final product were determined by MACSPlex Exosome kit. (d) Particle concentration. The average concentration (in 10^9^ particles/mL) of each sample was determined by NTA and by a correlation between NTA and MACPlex results. (e) Cryo‐EM analysis. Representative images of the final product depicting single‐ and multi‐bilayer bound vesicles. Scale bar = 100 nm. (f) ONi Super‐resolution Nanoparticle imaging. Representative images of a Triple‐Positive (TP) tetraspanin cluster identified within the final product. Individual CD9, CD63 and CD81 images and an overlay are presented. The count (%) for each cluster sub‐type (single, double or triple positive) for the final product is presented in the bar graph.

*In‐process and final product particle concentration and size distribution monitoring*: Particle concentration and particle size distribution analyses were performed by NTA (Table 3; Figure 3a,d). Both the mean and mode of particles size distributions remained within specifications (50 and 200 nm) throughout the manufacturing process; mean particle size was 128.2 nm in the spent media and 134 nm in the final product. The majority of particles detected were smaller than 200 nm. These particles constituted 79.9% of the total population in the spent medium and 82.3% after purification, concentration and sterilising filtration in the final product. This indicates that the particle profile was well preserved during the process.

*In‐process and final product identity monitoring—protein markers*: Particles were phenotyped by flow cytometry (MACSPlex Exosome Kit; Table 3; Figure 3b,c). The specifications established for the final product were MFI CD9 > 10, MFI CD63 > 10 and MFI CD81 > 10; the final product fulfilled these specifications (Table 3). These three tetraspanins were detected in spent media, and, as expected, their MFI expressions increased after TFF (Figure 3b).

Additional markers expressed on particles, as detected by flow cytometry using the MACSPlex Exosome Kit, are described in Figure 3c. Particles in the final product expressed CD133/1, CD326, CD29, CD41b, ROR1, SSEA‐4, CD24, CD49e, CD146, CD142, MCSP and CD44. The most expressed additional markers are CD133/1, CD326 and CD29.

*In‐process and final product particle quantification*: The MACSPlex Exosome Kit also enabled the calculation of particle concentration. This was possible due to a correlation established between CD9 MFI and NTA results for the present product (Wiklander et al. 2018). Using this flow cytometry method, particle concentration was found to increase during the manufacturing process, rising from 14 × 10^9^ particles/mL in spent media to 196 × 10^9^ particles/mL in the final product (Table 3 and Figure 3d). NTA alone and the correlation between NTA and MACSPlex Exosome Kit show the same trend: particle concentration is greatly increased following TFF, and this concentration is maintained through the sterilizing filtration step to the final product.

*Final product ultrastructural analysis*: The final product was analysed by Cryo‐EM. Morphology assessment revealed single, double and multiple bilayer bound vesicles (Figure 3e). Another microscopy technique based on tetraspanin staining (CD9, CD63 and CD81), ONi Super‐resolution Nanoparticle imaging, was also used to visualise the classical EV tetraspanins on the surface of each particle (Figure 3f). Particles identified in the final product were single, double, or triple positive for tetraspanins, with the most abundant sub‐type being CD63 single positive.

**FIGURE 4:**
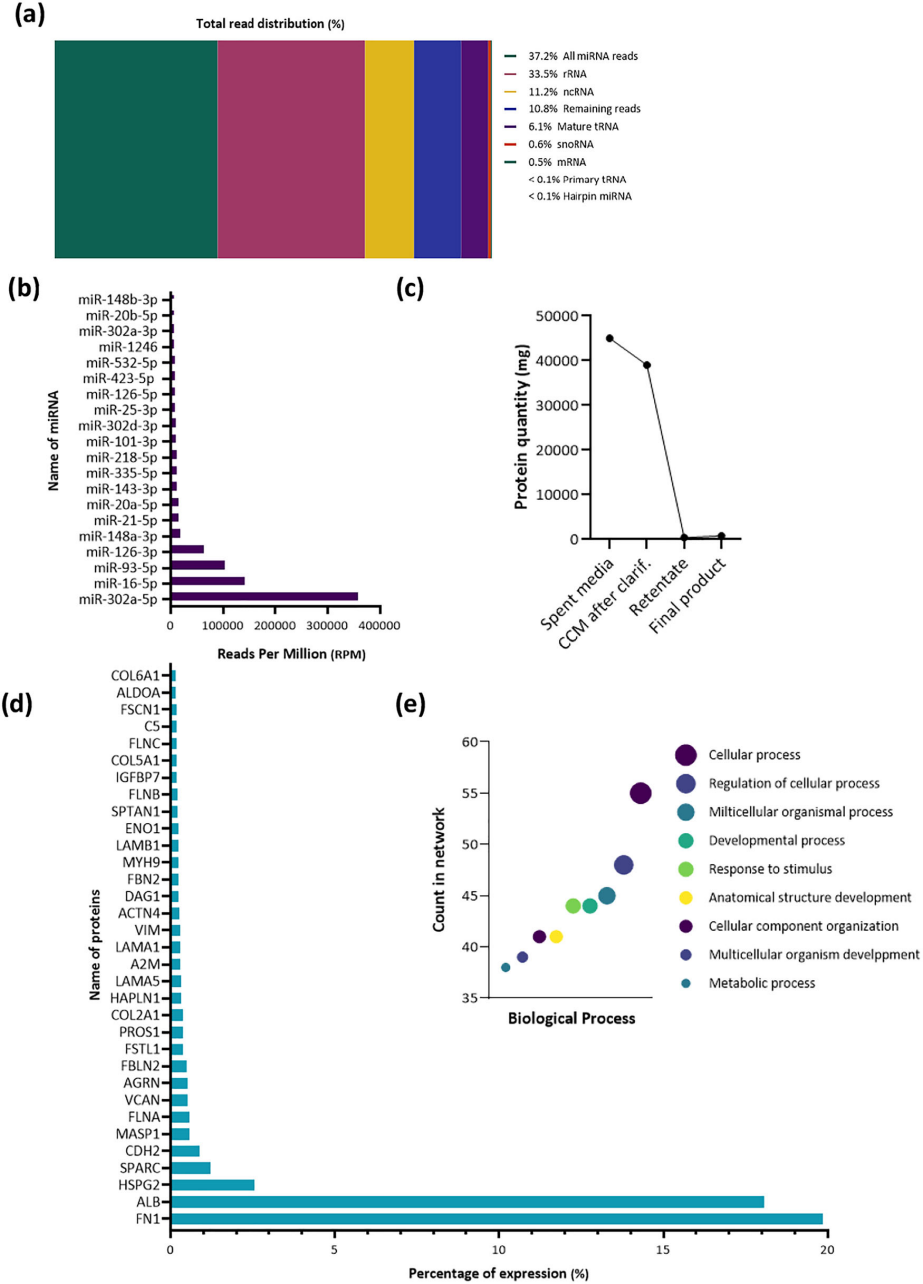
FIGURE 4 Characterisation of the EV‐enriched secretome composition. (a) Total read distribution (%) of different small RNA biotypes in the final product. Small RNA sequencing results were mapped to the human genome to determine the percentage of each RNA biotype. Sequences corresponding to all micro‐RNA (miRNA) reads, ribosomal RNA (rRNA), non‐coding RNA (ncRNA), remaining reads, mature transfer RNA (mature tRNA), small nucleolar RNA (snoRNA), mature micro‐RNA (mature miRNA), primary transfer RNA (primary tRNA) and hairpin micro‐RNA (hairpin miRNA) were identified. Note that the sequencing method is not optimal for identifying long reads, including mRNA. (b) Transcriptomics. The graph represents the top 20 most abundant miRNA identified in the final product, given as a percentage of expression (%). (c) Protein quantity. The total quantity of protein (mg) was measured in spent media, CCM after clarification, retentate and final product, using a BC Assay Kit. (d) Proteomics results. The most abundant proteins identified in the final product and their relative abundance (mass percentages) are shown. COL6A1, collagen alpha‐1(VI) chain; ALDOA, fructose‐bisphosphate aldolase A; FSCN1, Fascin; C5, complement C5 ; FLNC, filamin‐C; COL5A1, collagen alpha‐1(V) chain; IGFBP7, insulin‐like growth factor‐binding protein 7; FLNB, filamin‐B; SPTAN1, spectrin alpha chain, non‐erythrocytic 1; ENO1, alpha‐enolase; LAMB1, laminin subunit beta‐1; MYH9, myosin‐9; FBN2, fibrillin‐2; DAG1, dystroglycan; ACTN4, alpha‐actinin‐4; VIM, vimentin; LAMA1, laminin subunit alpha‐1; A2M, alpha‐2‐macroglobulin; LAMA5, laminin subunit alpha‐5; HAPLN1, hyaluronan and proteoglycan link protein 1; COL2A1, collagen alpha‐1(II) chain; PROS1, vitamin K‐dependent protein S; FSTL1, follistatin‐related protein 1; FBLN2, fibulin‐2; AGRN, agrin; VCAN, versican core protein; FLNA, filamin‐A; MASP1, mannan‐binding lectin serine protease 1; CDH2, cadherin‐2; SPARC, SPARC; HSPG2, basement membrane‐specific heparan sulphate proteoglycan core protein; ALB, albumin; FN1, fibronectin. (e) Gene ontology enrichment analysis in terms of biological process, analysed using String Prot.

*Final product nucleic acid profiling*: DNA and RNA content of the final product were also studied (Table 3; Figure 4). RNA sequencing found that the most abundant bio‐type by read number is the ‘all miRNA’ RNA bio‐type (Figure 4a). The most expressed miRNAs are detailed in Figure 4b. The top 3 included miR‐302a‐5p, miR‐16‐5p and miR‐93‐5p.

*Final product protein content assessment*: Protein concentration and quantity were assessed at each stage of the manufacturing process (Table 3; Figure 4c). Total protein quantity decreased from 45,000 mg in CCM after clarification to 10 mg in the TFF retentate (Figure 4c). As expected, the manufacturing process reduced the total protein amount, especially during the TFF step. Proteins contained in the final product were characterised by mass spectrometry. The proteomic analysis revealed a total of 1200 proteins (for which at least three peptides were identified). The 33 most abundant are described in Figure 4d. FN1 (fibronectin 1) was the most expressed, representing 20% of the total protein content and is derived from the cells as there is no fibronectin in the manufacturing process. This was confirmed by high expression levels of fibronectin mRNA in the CPC (*data not shown*). GO enrichment analysis was used to determine the implication of these proteins in biological processes (Figure 4e).

*Final product safety and purity*: Extractable volume in each glass tube was 1.96 mL on average (Table 3). No visible particles or colour deviations were observed (Table 3). The final product is sterile and exempt of mycoplasma and endotoxin. pH was 7.48 and within specifications according to EP 2.2.3 (Table 3).

*Final product purity*: Host cell protein quantification was assessed by measurement of GRP94, considered a preparation impurity, and was only slightly present at 122 ng/mL in the final product (Table 3).

### 3.4 In Vitro Functionality of the EV‐Enriched Secretome

*Final product, in vitro functional assessment*: The biological activity of the final product was evaluated by several in vitro functional assays: two characterisation assays (the cardiomyocyte survival assay, and the scratch wound healing assay) and one release assay (the HUVEC proliferation assay). Of the three in vitro functional assays, only the HUVEC proliferation assay was a qualified release assay with predefined release specifications.

**FIGURE 5:**
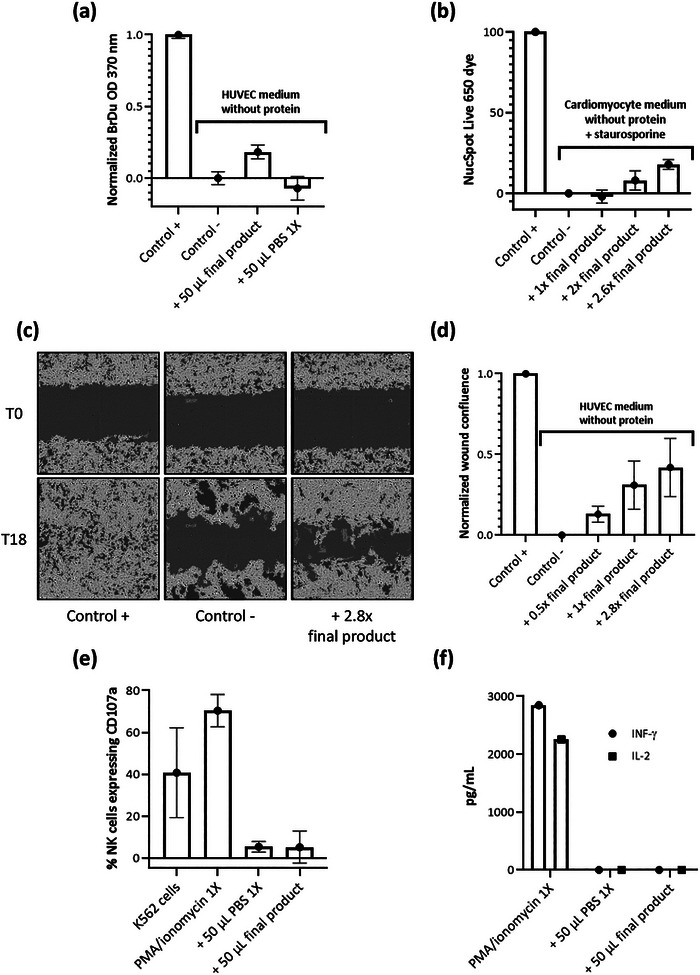
In vitro functional assays and immunogenicity assay. (a) Proliferation assay. HUVEC were plated and cultured in typical HUVEC media (unstressed, Control +) and in protein‐depleted media (stress condition). Stressed HUVEC were untreated (Control –) or treated with 50 µL of final product or 50 µL of PBS 1X (vehicle control). HUVEC proliferation was quantified by measuring BrdU uptake. Values were double normalised (baseline [negative control] subtracted, and normalised to the positive control). Data in the figure represent a single, representative experiment (average +/– SD; *n* = 5 technical replicates). (b) Cardiomyocyte survival assay. This test is a staurosporine‐induced cardiotoxicity assay. Cardiomyocytes were exposed to NucSpot Live 650 dye without staurosporine (Control +), with staurosporine alone (Control –) or with stauroporine and increasing concentrations of the final product. At the 24‐h time‐point, cell counts were determined by Incucyte. Values were double normalised (baseline [negative control] subtracted, and normalised to the positive control). Data in the figure represent a single, representative experiment (average +/– SD; *n* = 3 technical replicates). (c) Scratch wound healing assay. After establishing a monolayer of HUVEC, a scratch was induced using the Wound Maker according to the manufacturer's directions. Following the scratch, HUVEC were cultured in typical HUVEC media (unstressed, Control +) and in protein depleted (stress condition) media. Stressed HUVEC were untreated (Control –) or treated with final product. Plates were imaged by Incucyte at T0 and every 2 h up to 18‐h. Representative images taken on the Incucyte depicting individual wells are shown immediately after the scratch (T0) and 18 h post‐treatment (T18). Data in the figure represent a single, representative experiment. (d) Scratch wound healing assay. The results of HUVEC scratch wound healing assay described above were determined using the Scratch Wound Healing Module. Normalised wound confluence results at the 18‐h timepoint were double normalised (baseline [negative control] subtracted, and normalised to positive control). Data in the figure represent a single, representative experiment (average +/– SD; *n* = 5 technical replicates). (e) NK cell degranulation. The percent of human NK cells expressing CD107a was measured by flow cytometry. Human NK cells were cultured in the presence of human K562 cells (positive control), PMA/ionomycin 1X (second positive control) or 50 µL of PBS 1X (vehicle control) or 50 µL of final product. In presence of final product, there is a dramatic absence of expression of CD107a compared with the positive controls. Data in the figure represent a single, representative experiment (average +/– SD; *n* = 3 technical replicates). (f) PBMC alloreactive activation. The levels of multiple cytokines, IFN‐γ, and IL‐2 were measured by flow cytometry. Human PBMC were cultured in the presence of PMA/ionomycin 1X (positive control), 50 µL of PBS 1X (vehicle control) or 50 µL of the final product. Data in the figure represent a single, representative experiment.

*HUVEC proliferation assay results*: In the presence of 50 µL of final product, HUVEC proliferation, demonstrated by BrdU‐incorporation, increased by 47% compared to HUVEC treated with PBS 1X alone (vehicle control; *p* < 0.001) (Table 3; Figure 5a). This result met the pre‐defined criterion of >20% increase in proliferation, confirming the expected potency of the final product (Table 3). *Cardiomyocyte survival assay results*‐ In the presence of a 2.8× dose of our final product, staurosporine‐stressed cardiomyocytes showed a 30% increase in survival compared to controls (Figure 5b). *Scratch wound healing assay results*‐ In the presence of a 2.8× dose of the final product, would healing was increased by 40% compared to controls (Figure 5c,d).

*Final product, in vitro alloreactivity assessment*: In the presence of 50 µL of final product, human NK cells did not express significantly more CD107a compared to the control (5% of CD107a with final product, and 4.5% with PBS 1X) (Figure 5e). Likewise, human PBMC, when exposed to 50 µL of final product, did not secrete significantly more of the inflammatory cytokines IFN‐γ or IL‐2 compared to control (<3.2 pg/mL of IFN‐γ and <0.6 pg/mL of IL‐2 with both PBS 1X or final product) (Figure 5f).

### 3.5 Preclinical In Vivo Toxicity and Tumorigenicity

*Final product, in vivo toxicity assessment*: In Sprague Dawley male and female rats, the final product was administered in the tail vein (Figure S1a). The animals were sacrificed on Day 14. Body weight and food consumption did not differ between experimental and control groups during the follow‐up period (Figures S1b,c). At sacrifice, neither haematological, blood nor urinary biochemical parameters differed between experimental and control groups (Figure S1d–f). Organs were then collected. Their weights and histological analyses did not differ between experimental and control groups (Figure S1g; *histological data not shown*).

In Balb/c male and female mice, the final product was similarly administered intravenously with a sacrifice at Day 14 (Figure S2a). As in rats, at this time point, there were no differences between experimental and control groups in any of the parameters investigated (body weight and food consumption (Figure S2b,c), haematological and biochemical parameters in blood (Figure S2d,e), organ weights and histological features (Figure S2f)).

*Final product, in vivo tumorigenicity assessment*: Tumorigenicity assessment was performed in nude mice following a single SC administration of the final product (Figure S3a). *Positive control (HeLa cells) group*: All male and female animals given HeLa cells developed at least two masses during the follow‐up period, with up to four masses in some animals, prior to sacrifice. For reasons of animal welfare, all positive control group animals were prematurely euthanised; one animal was euthanised on Day 8, and all remaining animals were euthanised on Day 13. *Final product and vehicle control (PBS 1X) group*: No mouse injected with PBS 1X (negative control) or the final product developed any tumour, and all survived throughout the 91 days of follow‐up.

At sacrifice (Day 8/13 for HeLa group, or day 91 for the control and test groups), there were no differences between experimental and control groups in any of the parameters investigated (body weight [Figure S3b], haematological and clinical chemistry parameters [Figure 3c,d] and organ weight (Figure S3e)). No macroscopic abnormalities were observed in mice receiving the final product, the vehicle control or the positive control except for in the spleen in the female group given HeLa cells. Any small differences between test item‐treated groups and controls were interpreted as incidental and/or within the normal range of variation, except for the spleen, where the anomalies detected in the female HeLa group were attributed to an immune reaction against HeLa cells.

### 3.6 Stability of the Final Product

**FIGURE 6:**
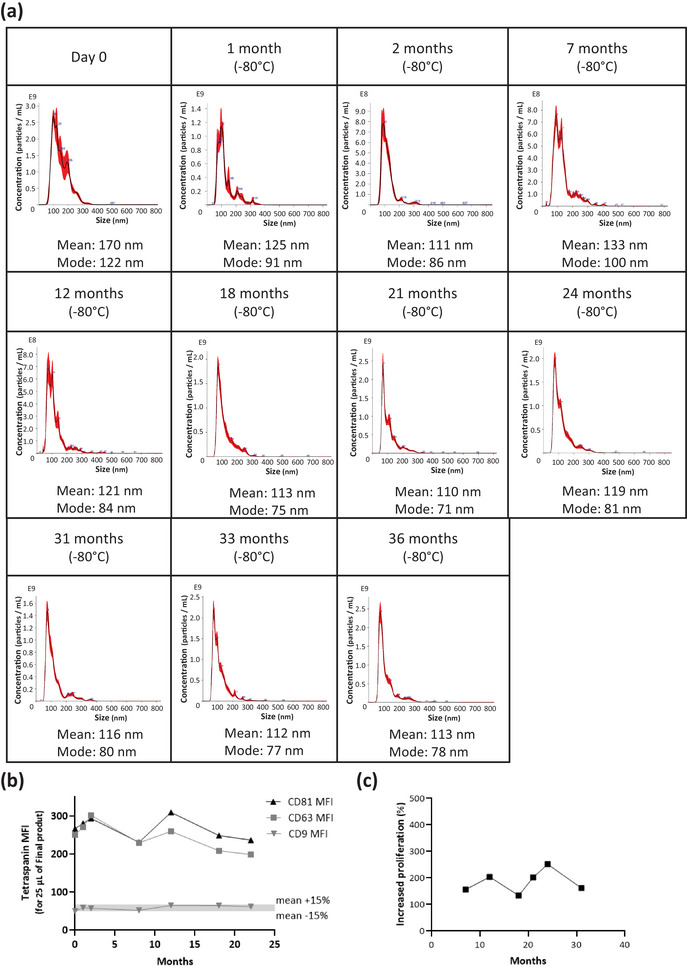
Long term stability (up to 36 months of storage). (a) Particle size distribution with mean and mode particle size in the final product, by NTA, over time. (b) Tetraspanin marker expression (CD81, CD63 and CD9) in final product by MACSPlex Exosome kit, over time. (c) HUVEC proliferation assay results for final product, over time.

For long‐term storage, the final product was stored between –65°C and –85°C and its stability was assessed approximately every 3–6 months over a 3‐year period (Figure 6). The study is ongoing and will continue until the end of the clinical trial. So far, stability studies have found that the final product has remained sterile and the glass tubes intact after 36 months (*data not shown*). Particle size distribution has remained within specifications (acceptable range: 50–200 nm) over the 36 months of stability testing (Figure 6a). Mean and mode particle sizes as well as CD81, CD63 and CD9 expression levels, and relative expression profiles, have remained stable over the testing period (Figure 6b). CD9 MFI variability did not exceed ±15% over time, as required by EP 2.9.6. (Figure 6b). The final product also retained its potency in the HUVEC proliferation assay, within tolerances (Figure 6c).

## 4 Discussion

EV‐enriched secretomes may represent a novel class of therapeutics for a large variety of diseases (Uddin et al. 2024). However, a number of practical issues must first be addressed to provide straightforward access to these acellular products, which includes establishing controlled, scalable, GMP‐compliant manufacturing and QC release methods that are acceptable to the regulatory agencies. To our knowledge, the present study is the first to describe the development and success of a GMP process for the manufacture and release of an EV‐enriched secretome from an hiPSC‐derived differentiated cell‐type that has been authorised for use in a human clinical trial.

In this article, we have described a novel process meeting the above criteria for the manufacture of a CPC‐derived EV‐enriched secretome product. One important step was the incorporation of sterilising filtration. This step was included to align with current regulatory requirements for biological products, and for improved patient safety assurance. For our product, functionality tests before and after 0.22 µm filtration showed no significant impact on therapeutic efficacy in vitro. However, we acknowledge that this may not be true for all secretome products; in some cases, sterilising filtration could eliminate key therapeutic components, such as large vesicles, from the final composition. For such products, alternative methods may be required to ensure sterility while maintaining efficacy.

We have also described here a novel QC strategy which confirmed process control, and final product identity, purity, safety and in vitro efficacy. Particular attention was paid to the potency criterion, as this is an important gatekeeper of product release for clinical use and is critically scrutinised by the regulators as part of the risk analysis. Potency assays should measure the biological activity of the material and be suitably linked to the expected biological properties of the therapy (Gimona et al. 2021). Defining relevant assay and quality metrics is, however, challenging in the case of secretomes because the mechanisms of action of EV are not yet fully mapped and are likely multifaceted. In our case, one of the presumed effects of the EV is to improve the fitness of their target cells. A proliferation assay was therefore selected as the main potency assay for product release and stability testing. Human endothelial cells were selected as the in vitro target cells, as these are a relevant cell type in blood vessels and lymphatic tissues (which are involved in the complex pathology cardiovascular disease). Further, these cells are easily cultured in the lab in a standardised way, and are readily commercially attainable. The endothelial cell proliferation assay was then designed to assess product quality in a straight‐forward and quantitative way so as to enable assay qualification. The potency assay was successfully qualified according to ICHQ2 guidelines, and the final product met the pre‐specified efficacy threshold (Table 3). In addition, as there is also compelling evidence that EV originating from CPC have angiogenic and anti‐apoptotic effects, two additional, for‐information‐only (FIO), assays were employed for further product characterisation: the endothelial cell scratch wound healing assay, and a novel cardiomyocyte survival assay. Both assays were used throughout process/product development, and to characterise the final, clinical batch. The IMP was found to have positive effects in both assays, further supporting the multifaceted therapeutic nature of the EV‐enriched secretome product.

An additional in vitro assay, an alloreactivity assessment, was employed as part of the QC release strategy to evaluate product safety. The IMP was, indeed, found to be non‐immunogenic in vitro.

**FIGURE 7:**
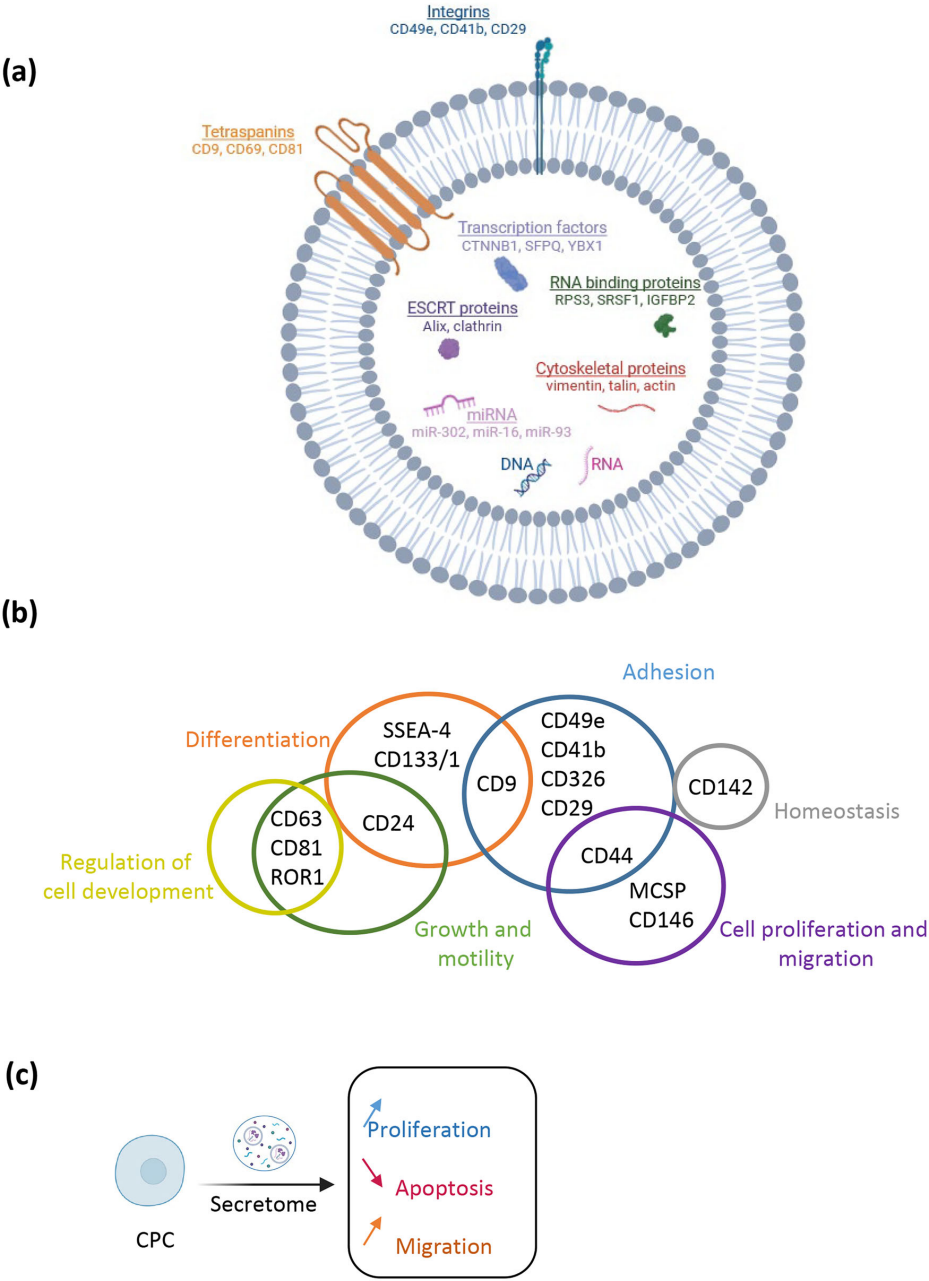
Summary of the components and biological effects of the EV‐enriched secretome final product. (a) Some of the protein and molecular components identified in the final product. Image created with BioRender.com. (b) Summary of cell surface markers identified by the MACSPlex Exosome kit in the final product, which their known roles in biological processes. (c) Functional effects of the final product. Image created with BioRender.com.

The molecular components of the IMP have also been characterised, with particular attention paid to the small EV component (Figure 7a). Their presence was confirmed by Cryo‐EM demonstrating lipid‐bilayer‐bound objects of appropriate size and structure. The nature of these EV was consistent with expectations, including a size range of particles from 20 to 200 nm by NTA and the presence of all three tetraspanin markers of EV (CD9, CD63 and CD81; Théry et al. 2018), as evidenced by flow cytometry and ONi Super‐resolution Nanoparticle imaging. In addition, to quantify the particles, we developed and qualified a counting method based on a correlation between NTA measurements and the MFI of CD9 expression, as detected by the MACSPlex exosome Kit for flow cytometry. This metric overcomes some of the limitations of NTA and meets the regulatory expectations of an analytical method.

The RNA and protein content of the product were characterised, and the results suggest that these cargoes could be contributing to some biological functions relevant to heart repair. Namely, among the top 20 most abundant miRNAs identified in the final product, miR‐302a, miR‐16‐5p and miR‐93‐5p, which represented 36%, 14% and 10%, respectively, of all the expressed miRNAs, have been involved in: the reduction of post‐infarction scar formation (Tian et al. 2015) and vascular inflammation (Yuan et al. 2021); the suppression of activation of inflammatory macrophages (Liang et al. 2016); and the prevention of cardiac damage by inhibition of autophagy and inflammation (Liu et al. 2018).

Similarly, many of the proteins identified in the product are associated with biological processes that may contribute to tissue repair (Figure 7b). Of note, one of these proteins present in the cargo, follistatin, has recently been reported to stimulate pig cardiomyocyte proliferation (Wei et al. 2025). Figure 7c presents a schematic summarising some of the key biological processes presumed to be modulated by the secretome preparations.

Tumorigenicity and toxicity are critical risk factors that also have to be integrated in the risk analysis before clinical application. Results in GLP mouse and rat studies showed that the secretomes did not induce signs of toxicity, and neither did they promote the development of tumours.

A novel stability program was developed and approved by the regulatory agency, including assessment of particle concentration and tetraspanin profiles for identity and purity, sterility for safety, and HUVEC proliferation assay for assurance of biological activity. The product was found to be stable between –65°C and –85°C for at least 3 years. As such, it remains readily available for off‐the‐shelf, allogeneic use, and has been used in the ongoing clinical trial.

More work remains to be done in the field, such as increasing product concentration while maintaining its solubility, improving stability and bioavailability (Rayaprolu et al. 2018), developing more robust and qualifiable analytical platforms (equipment, assays, standards, calibrators and controls; Welsh et al. 2015, 2021), understanding mechanisms of action (Lener et al. 2015; Barile and Vassalli 2017; Reiner et al. 2017), characterising pharmacokinetics, and manipulating natural biodistribution patterns to enhance organ‐specific targeting following systemic delivery. However, the work here described is yet significant in several ways.

First, we have demonstrated that, despite multiple material, reagent and process changes from the original research‐grade experiments, we could develop a cost‐effective process which generates a GMP‐grade material that remained efficacious in vitro and in animal models of the target disease.

Second, we have developed an in‐process and QC release strategy which combines regulatory guidelines from several different, albeit, relevant types of therapy; this strategy was necessary since no European guidelines yet exist which are truly specific to an EV‐enriched secretome product.

Third, our overall strategy focusses on controlling the key factors that can influence batch‐to‐batch variability considering both the mother cells (CPC) and the final product. A critical aspect of controlling the mother cells is that each batch of CPC is generated from a single bank of hiPSC, derived from a single donor. This is a major advantage over other, primary‐cell dependent therapies, since it eliminates the potential of donor‐to‐donor variably. This variability has been a key contributor to the difficulties of bringing primary MSC and primary MSC‐EV therapies to market. Several additional process parameters were also controlled, such as CPC seeding density, media composition, cell handling and incubation time and temperature. These steps could be optimised and adhered to, since, again, each batch initially started with the exact same hiPSC material. QC criteria were then developed to monitor batch reproducibility and ensure that each batch meets predefined specifications for cells, spent media and final product. This multilayered quality assurance approach was successful, with excellent batch‐to‐batch consistency found between pre‐clinical batches and our clinical batch (Table S1), reinforcing the robustness of the manufacturing process, and supporting the clinical applicability of our EV‐based product.

Third, the manufacturing method, in‐process monitoring and QC release strategy presented here are directly amenable to the large scale, GMP manufacturing of EV‐enriched secretome products from other cell types (including, MSC) and should thus be viewed as an integrated platform applicable to indications extending beyond the field of cardiology. Specifically, our approach of limiting the protein content in the vesiculation media will reduce the non‐secretome components in a final product, and the use of in‐line filtration and large‐scale TFF for concentration/isolation can be applied to the GMP manufacture of secretomes from other cell types, and at scales appropriate for early phase clinical trials up to commercial manufacturing scale.

The processes described here for the manufacture, characterisation and quality assurance of the EV‐enriched secretome from hiPSC‐derived CPC were successful in producing an EV‐based IMP which has been thoroughly evaluated by the competent regulatory authority and approved for the Phase I human clinical trial which is currently ongoing (SECRET‐HF). The clinical protocol details and results for the first patient have been published (Menasché et al. 2024). Together with the efforts of other pioneering teams in the EV field (Lamparski et al. 2002; Andriolo et al. 2018; Busatto et al. 2018; Rohde et al. 2019), these results are helping to pave the way for the advancement of EV‐based therapeutics.

## Author Contributions

C.H. and G.C. contributed to process development, designed experiments, analysed the data, contributed to regulatory compliance and wrote the manuscript. C.C. performed QC release testing. I.D. performed IMP manufacturing. M.H. and J.W. contributed to process development, CPC manufacture and release, designed performed and analysed some of the in vitro biological activity experiments. M.H., J.W. and K.B. contributed to transcriptomic analyses. V.B. contributed to process development and EV characterisation. J.F. performed residual DNA analyses. F.D. and D.L. performed proteomic analyses. J.L. contributed to clinical trial design. J.‐R.F. contributed to regulatory compliance strategy and experimental design. P.M. contributed to program leadership, process development, analytical strategy, regulatory compliance and clinical trial design, experimental design, data analysis and drafting of the manuscript. N.K.R. contributed to process development, experimental design, data analysis and drafting of the manuscript. All authors reviewed the manuscript.

## Conflicts of Interest

Michele Hamrick, Kiranmayee Bakshy and Nisa K. Renault are a full‐time employees of FUJIFILM Cellular Dynamics Inc. At the time of the work, Jacquelyn Wong was a full‐time employee of FUJIFILM Cellular Dynamics, Inc. No other author declares a conflict of interest. This work is the subject to one or more provisional and PCT patents applications.

## Supporting information




**Supporting Fig. 1**: In vivo toxicity studies.

**Supporting Fig. 2**: In vivo toxicity studies.

**Supporting Fig. 3**: In vivo tumorigenicity studies.

**Supporting Fig. 4**: jev270145‐sup‐0004‐TableS1.docx

**Supporting Fig. 5**: jev270145‐sup‐0005‐Supp‐figures‐legend.docx

## Data Availability

The data that support the findings of this study are available from the corresponding author upon reasonable request.
