# Supplementary material for: GMP‐Compliant Process for the Manufacturing of an Extracellular Vesicles‐Enriched Secretome Product Derived From Cardiovascular Progenitor Cells Suitable for a Phase I Clinical Trial
Source: J Extracell Vesicles. 2025 Aug 20;14(8):e70145. doi: 10.1002/jev2.70145 (PMC12365392; doi:10.1002/jev2.70145)

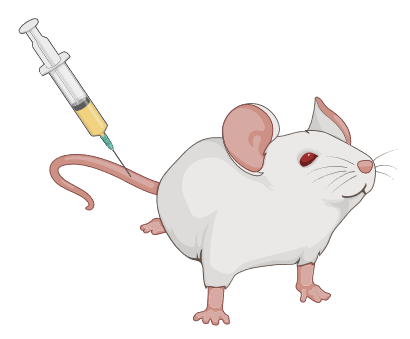


0 d

14 d

In-life observations /

measurements

End of study

Three IV administrations

Final product or

vehicle control (PBS 1X)

Mice

**(a)**

**Supplementary FIGURE 2**

**(c)**

**(b)**

**(d)**

**(e)**

**(f)**


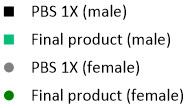

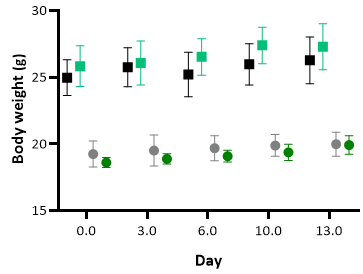

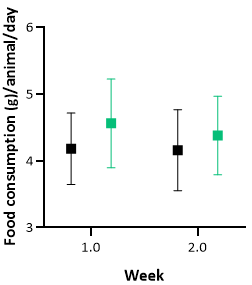

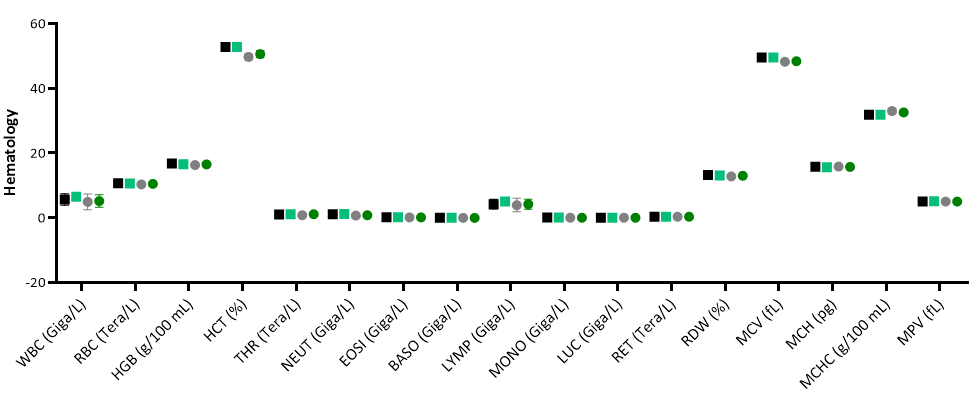

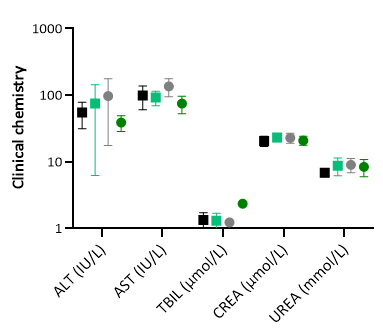

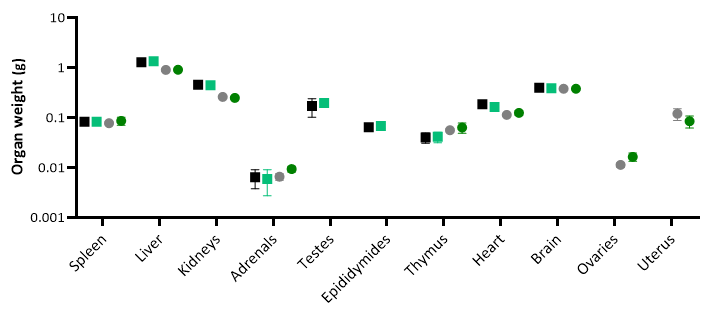

Supplement: Supplementary file 2 — Supporting Fig. 2: In vivo toxicity studies. [file JEV2-14-e70145-s005.docx]
