# Supplementary material for: GMP‐Compliant Process for the Manufacturing of an Extracellular Vesicles‐Enriched Secretome Product Derived From Cardiovascular Progenitor Cells Suitable for a Phase I Clinical Trial
Source: J Extracell Vesicles. 2025 Aug 20;14(8):e70145. doi: 10.1002/jev2.70145 (PMC12365392; doi:10.1002/jev2.70145)

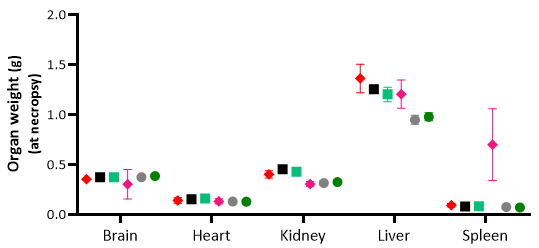

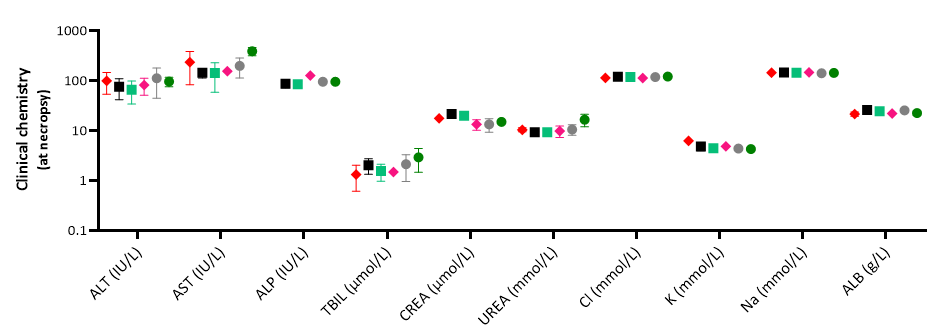

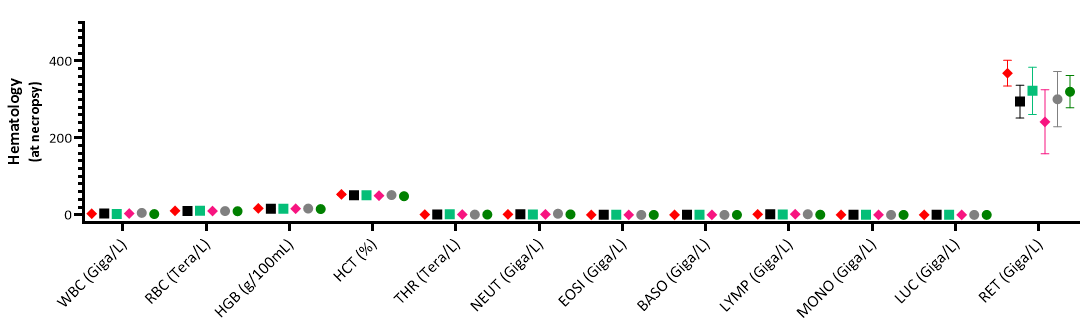

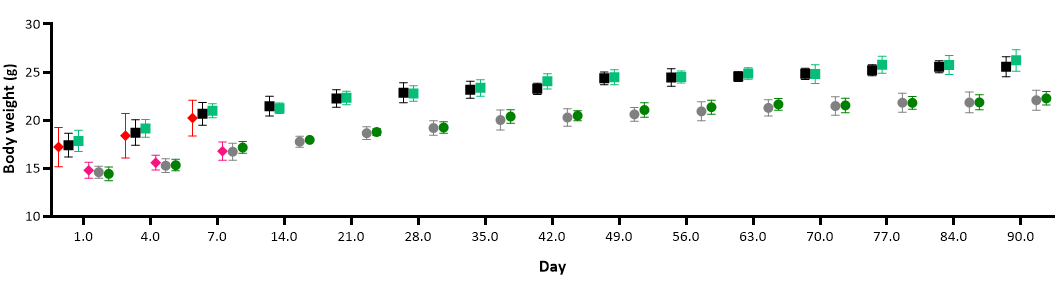


**Supplementary FIGURE 3**

**(b)**

**(d)**

**(e)**

**(c)**


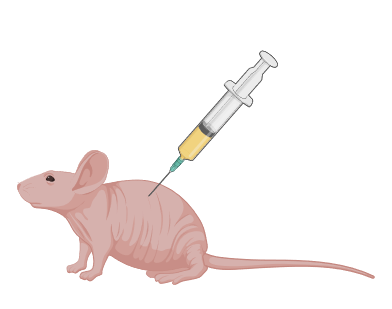


Mice

Final product or

vehicule control (PBS 1X) or positive control (HeLa cells)

0 d

91 d

In-life observations /

measurements

End of study

One SC administration

**(a)**

*

**

*

**

**One HeLa animal euthanized*

**All remaining HeLa animals euthanized*


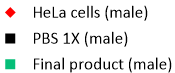

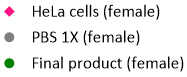

Supplement: Supplementary file 3 — Supporting Fig. 3: In vivo tumorigenicity studies. [file JEV2-14-e70145-s001.docx]
