# Supplementary material for: GMP‐Compliant Process for the Manufacturing of an Extracellular Vesicles‐Enriched Secretome Product Derived From Cardiovascular Progenitor Cells Suitable for a Phase I Clinical Trial
Source: J Extracell Vesicles. 2025 Aug 20;14(8):e70145. doi: 10.1002/jev2.70145 (PMC12365392; doi:10.1002/jev2.70145)
Supplement: Supplementary file 4 — Supporting Fig. 4: jev270145‐sup‐0004‐TableS1.docx [file JEV2-14-e70145-s002.docx]

***Supplementary Table 1. Batch-to-batch consistency of CPC during vesiculation*** (Parameter, Method, Specifications, Results).

| **Parameter** | ***Method*** | ***Specifications*** | ***Results: pre-batch*** | | ***Results: clinical batch*** | |
| --- | --- | --- | --- | --- | --- | --- |
|  |  |  | ***Day+3*** | ***Day+5*** | ***Day+3*** | ***Day+5*** |
| **Number of**  **viable cells** | NucleoCounter® NC-200™  (DAPI / AO staining) (EP 2.7.29) | Informative | 7.9 x 10^9^ | 6.5 x 10^9^ | 9.5 x 10^9^ | 8.1 x 10^9^ |
| **% viability** |  | > 70 | 84.0 | 93.0 | 91.5 | 91.5 |
| **Microbiological**  **sterility** | Bact / Alert®  (EP 2.6.1 and 2.6.27) | Negative at 10 days | Negative at 10 days | Negative et 10 days | Negative et 10 days | Negative et 10 days |
| **Mycoplasma** | qPCR (EP 2.6.7) | < 10 CFU/mL | < 10 CFU/mL | < 10 CFU/mL | < 10 CFU/mL | < 10 CFU/mL |
| **Endotoxin** | Kinetic chromogenic LAL  (EP 2.6.14) | < 2 EU/mL | < 2 EU/mL | < 2 EU/mL | < 2 EU/mL | < 2 EU/mL |
| **Karyotype** | RHG-banding | No abnormalities | / | No abnormalities | / | No abnormalities |
| **Identity** | Flow Cytometry  (EP 2.7.24) | < 5% SOX2/NANOG  > 95% CD56/CXCR4 | < 5% SOX2/NANOG  > 95% CD56/CXCR4 | < 5% SOX2/NANOG  > 95% CD56/CXCR4 | < 5% SOX2/NANOG  > 95% CD56/CXCR4 | < 5% SOX2/NANOG  > 95% CD56/CXCR4 |
|  |  | cTNT MFI : increasing | 10.5 | 12.9 | 6.3 | 12.3 |
|  |  | αMHC MFI: increasing | 27.2 | 85.5 | 37.7 | 46.3 |
| **Residual**  **hiPSC testing** | ddPCR | < 0.37 copies/µL (corresponding to < 2% of residual hiPSC) | Below detectable limits  (i.e., not detectable) | Below detectable limits  (i.e., not detectable) | Below detectable limits  (i.e., not detectable) | Below detectable limits  (i.e., not detectable) |

*DAPI: 4′,6-diamidino-2-phenylindole; AO: Acridine Orange; EP: European Pharmacopeia; qPCR: quantitative real-time PCR; CFU: Colony Forming Unit; LAL: Limulus Amebocyte Lysate; EU: Endotoxin Unit; MFI: Median Fluorescence Intensity; cTNT: cardiac Troponin T;* α*MHC: alpha Myosin Heavy Chain; ddPCR: digital droplet PCR; hiPSC: human induced Pluripotent Stem Cell.*
