# Supplementary material for: GMP‐Compliant Process for the Manufacturing of an Extracellular Vesicles‐Enriched Secretome Product Derived From Cardiovascular Progenitor Cells Suitable for a Phase I Clinical Trial
Source: J Extracell Vesicles. 2025 Aug 20;14(8):e70145. doi: 10.1002/jev2.70145 (PMC12365392; doi:10.1002/jev2.70145)
Supplement: Supplementary file 5 — Supporting Fig. 5: jev270145‐sup‐0005‐Supp‐figures‐legend.docx [file JEV2-14-e70145-s003.docx]

**Supplementary FIGURE 1**

In vivo toxicity studies.

1. Toxicity study in immunocompetent rats. Final product or vehicle control (PBS 1X) was IV administered in the tail vein. In-life and end point measurements included: morbidity/mortality, clinical observations, body weight, food consumption, clinical chemistry, hematology, organ weight, and macroscopic and microscospic observations. Image created with BioRender.com.
2. Body weight. Homogeneity of groups was validated on the criterion of body weight (in g) measured on day 0, separately for males and females. Body weight results (average and standard deviation) are presented with a jitter at each time point.
3. Food consumption per animal and per day. Food consumption (in g)/animal/day was measured weekly for each group. Averages are presented as a scatterplot with a jitter at each time point.
4. Hematological parameters: WBC= white blood cell count; RBC= red blood cell count; HGB= haemoglobin; HCT= haematocrit; THR= thrombocyte count; NEUT= neutrophil count; EOSI= eosinophil count; BASO= basophil count; LYMP= lymphocyte count; MONO= monocyte count; LUC= large unstained cell count; RET= reticulocytes; RDW= red distribution width; MCV= mean corpuscular volume; MCH= mean corpuscular haemoglobin; MCHC= mean corpuscular haemoglobin concentration; MPV= mean platelet volume; APTT= activated partial thromboplastin time; PT = prothrombin time. Hematology parameters were determined on the day of necropsy.
5. Clinical chemistry parameters: ALT= alanine aminotransferase; AST= aspartate aminotransferase; ALP= alkaline phosphatase; TBIL= total bilirubin; PROT= total protein; CREA= creatinine; UREA= urea nitrogen; GLU= glucose; CHOL= total cholesterol; Cl= chloride; K= potassium; Na= sodium; ALB= albumin; TRIG= triglycerides; Ca= calcium; P= phosphorus; GLOB= globulin; A/G= ratio albumin/globulin. Clinical chemistry parameters were determined on the day of necropsy.
6. Urinalysis. Urine was collected from all animals one day prior to necropsy. Urine collection was performed individually in metabolism cages overnight.
7. Organ weight, on the day of necropsy.

**Supplementary FIGURE 2**

In vivo toxicity studies.

1. Toxicity study in immunocompetent mice. Final product or vehicle control (PBS 1X) was IV administrated in the tail vein. In-life and end point measurements included: morbidity/mortality, clinical observations, body weight, food consumption, clinical chemistry, hematology, organ weight, and macroscopic and microscospic observations. Image created with BioRender.com.
2. Body Weight. Homogeneity of groups was validated on the criterion of body weight (in g) measured on the day 0, separately for males and females. . Body weight results (average and standard deviation) are presented with a jitter at each time point.
3. Food consumption per animal and per day. Food consumption (in g)/animal/day was measured weekly for each group. Data for females is not available. Averages are presented as a scatterplot with a jitter at each time point.
4. Hematological parameters. Hematology parameters were performed on the day of necropsy.
5. Clinical chemistry parameters. Clinical chemistry parameters were determined on the day of necropsy.
6. Organ weight, on the day of necropsy.

**Supplementary FIGURE 3**

In vivo tumorigenicity studies.

1. Tumorigenicity study in nude mice. Final product, vehicle control (PBS 1X) or positive control (HeLa cells) were administered once on day 0 subcutaneously. In-life and end point measurements included: morbidity/mortality, clinical signs, body weight, food consumption, clinical chemistry, hematology, organ weight, macroscopic and microscospic observation; and histopathology. For reasons of animal welfare, all animals in the HeLa group were prematurely euthanized. Each HeLa injected animal developed at least two masses during the follow-up period, with some developing up to four masses prior to euthanasia. One animal was euthanized on day 8 (star), and all remaining HeLa group animals were sacrificed on day 13 (double star). Image created with BioRender. com.
2. Body weight. Animals were allocated to groups based on day 0 body weights such that groups had approximately equal initial mean body weights. Body weight results (average and standard deviation) are presented with a jitter at each time point. Note that no data are collected for the HeLa group beyond day 13 as all animals in that group were prematurely euthanized.
3. Hematological parameters. Hematology parameters were performed on the day of necropsy.
4. Clinical chemistry parameters: Clinical chemistry parameters were determined on the day of necropsy.
5. Organ Weight, on the day of necropsy.
